# Linearization routines for the parameter space concept to determine crystal structures without Fourier inversion

**DOI:** 10.1107/S1600576725001955

**Published:** 2025-05-23

**Authors:** Muthu Vallinayagam, Melanie Nentwich, Dirk C. Meyer, Matthias Zschornak

**Affiliations:** a Technical Physics, University of Applied Sciences, Friedrich-List-Platz 1, 01069 Dresden, Germany; b Center for Efficient High Temperature Processes and Materials Conversion ZeHS, TU Bergakademie Freiberg, Winklerstr. 5, 09596 Freiberg, Germany; c Deutsches Elektronen-Synchrotron DESY Notkestr 85 22607 Hamburg Germany; d Institute of Experimental Physics, TU Bergakademie Freiberg, Leipziger Str. 23, 09596 Freiberg, Germany; DESY, Hamburg, Germany

**Keywords:** crystal structure determination, centrosymmetric structures, two- and three-dimensional parameter space, linearization, parameter space concept, Monte Carlo simulation, one-dimensional projection, X-ray diffraction

## Abstract

We present the elaboration and first generally applicable linearization routines of the parameter space concept (PSC) for determining one-dimensionally projected structures of *m* independent scatterers. This crystal determination approach does not rely on Fourier inversion but rather considers all structure parameter combinations consistent with available diffraction data in a parameter space of dimension *m*. The method utilizes *m* structure-factor amplitudes or intensities represented by piecewise analytic hyper-surfaces to define the acceptable parameter regions. The coordinates of the point scatterers are obtained through the intersection of multiple isosurfaces. This approach allows for the detection of all possible solutions for the given structure-factor amplitudes in a single derivation. Taking the resonant contrast into account, the spatial resolution achieved by the presented method may exceed that of traditional Fourier inversion, and the algorithms can be significantly optimized by exploiting the symmetry properties of the isosurfaces. The applied one-dimensional projection demonstrates the efficiency of the PSC linearization approach based on fewer reflections than Fourier sums. Monte Carlo simulations, using the projections of various random two- and three-atom structure examples, are presented to illustrate the universal applicability of the proposed method. Furthermore, ongoing efforts aim to enhance the efficiency of data handling and to overcome current constraints, promising further advancements in the capabilities and accuracy of the PSC framework.

## Abstract 2

Novel linearization routines within the parameter space concept (PSC) provide an alternative approach to determine one-dimensionally projected crystal structures from a few diffraction intensities of standard Bragg reflections, represented by piecewise analytic hyper-surfaces, without the use of Fourier inversion. By the intersection of linearized isosurface segments of multiple reflections, PSC accurately pinpoints the atomic coordinates and explores both homometric and quasi-homometric solutions in a single analysis.

## 1. Introduction

Solving crystal structures from diffraction intensities plays a vital role in many areas of solid-state research, *e.g.* physics, chemistry, mineralogy, materials sciences, biology and pharmacy, as it forms the fundamental basis for understanding the properties of materials as well as their effects and functionalities. The corresponding databases grow by tens of thousands of structures every year. The state-of-the-art structure determination methodology is based on Fourier inversion (FI) of the scattering density (*e.g.* electron density for X-ray diffraction, nuclear density for neutron diffraction). In the early days, the developments in crystallography were mainly based on computationally efficient FI techniques either directly or indirectly, such as the charge flipping method (Oszlányi & Süto, 2004 ▸), the algebraic method (Rothbauer, 1994 ▸; Rothbauer, 1995 ▸; Rothbauer, 1998 ▸), geometrical methods (Navaza & Silva, 1979 ▸), analytical function methods (Cervellino & Ciccariello, 2005 ▸), fit methods such as Rietveld refinement (Toby, 2024 ▸) and matching learning algorithms (Shi, 2022 ▸; Munteanu *et al.*, 2024 ▸; Billinge & Proffen, 2024 ▸).

The currently used FI techniques to construct electron density systematically introduce noise and errors in the calculation due to series termination. Therefore, a large number of terms in the FI series are required, which in turn necessitates a substantial set of experimental observations. Furthermore, the quality of X-ray diffraction intensities greatly influences the FI series, with weaker observations contributing less. However, the experimental database is in most cases incomplete since only the quadratic amplitudes of the Fourier coefficients (*i.e.* the structure factors via the reflection intensities) can be determined, and the well known phase problem of crystallography makes solving the structure more challenging (Harrison, 1993 ▸; Fischer *et al.*, 2005 ▸; Fischer *et al.*, 2008 ▸; Kirfel & Fischer, 2009 ▸).

**Figure 1:**
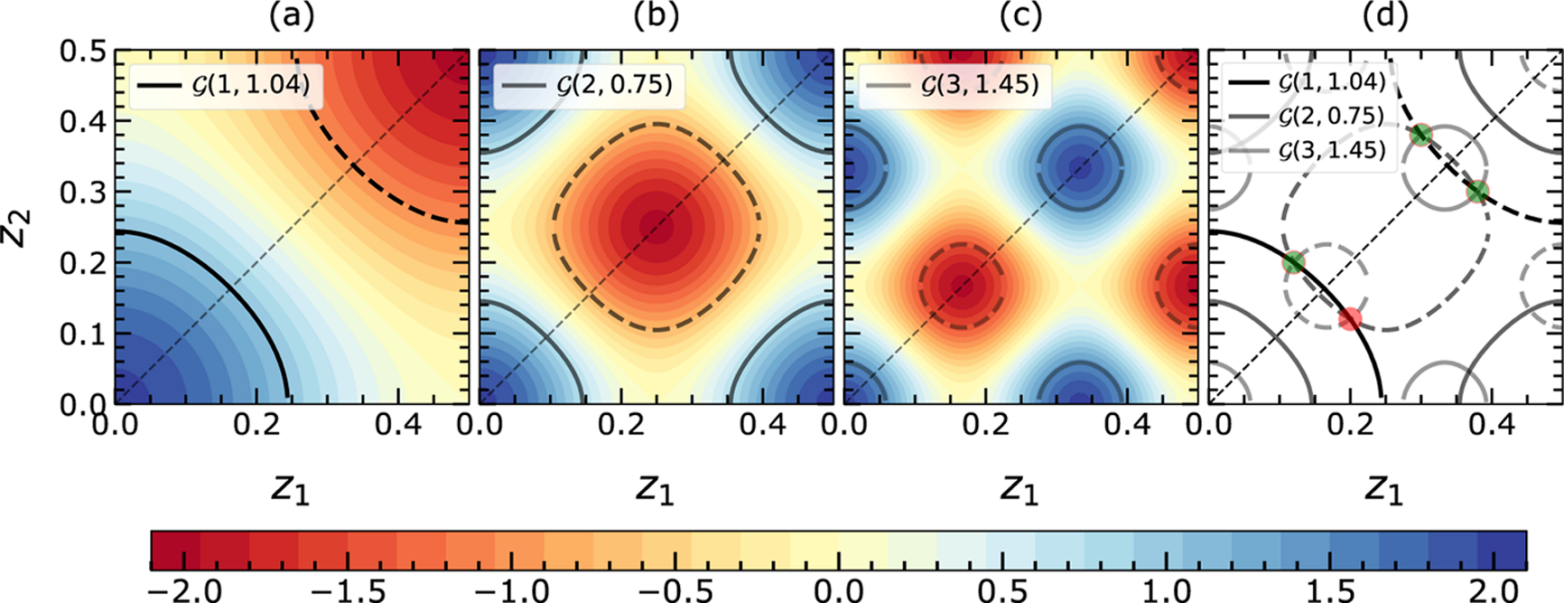
Basic explanation of the parameter space concept. (*a*)–(*c*) Two-dimensional parameter space for the projection of a crystal structure of two equal scatterers onto the *z* axis for reflections 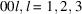. The color map represents the calculated amplitude for each combination of atomic coordinates. The isosurfaces for positive and negative amplitudes generated by the arbitrary atomic coordinates 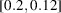 are highlighted with solid and dashed lines, respectively. (*d*) Overlay of the isosurfaces from (*a*)–(*c*) with their intersecting points highlighted by red and green circles. The intersection point at the red circle is the intended structure to be found, and alternative solutions are at the green circles.

In order to overcome the demerits of FI techniques, alternative methods have been developed. In this study, we examine the relationship between the structure factor and the atomic positions in crystal structures by considering the geometrical correlations. In general, an *m*-atom structure has 3*m* free positional parameters to be determined, which include the *x*, *y* and *z* coordinates of all *m* atoms. To simplify the structure-solving process with the parameter space concept (PSC), the task is split into several independent one-dimensional projections (in real space), each providing the solution of *m* parameters within an *m*-dimensional parameter space (PS; space of atomic coordinates with the orthogonal basis in 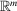) (Zschornak *et al.*, 2024 ▸; Fischer *et al.*, 2005 ▸; Knop, 1989 ▸; Pilz, 1996 ▸; Ott, 1928 ▸). Each point in the PS corresponds to a possible combination of atomic coordinates (*e.g.* projected onto the *z* axis) and generates a unique X-ray diffraction intensity for a predefined reflection. Vice versa, the set of points that reproduce the experimentally observed intensity of a particular reflection defines a manifold called an isosurface [see Figs. 1 ▸(*a*)–1 ▸(*c*)]. The intersection point from all isosurfaces of different reflections expresses the intended structure [see Fig. 1 ▸(*d*)].

The theory of PSC has been developed during the past two decades, mainly focusing on equal atoms (Knop, 1989 ▸; Pilz, 1996 ▸; Ott, 1928 ▸), aiming to achieve higher spatial resolution than the FI techniques while at the same time using a reduced number of available intensity data sets. Apart from these advantages, PSC will always recognize all possible solutions that can reproduce the given experimental intensities, although at the cost of parameterizing functions in continuous high-dimensional spaces. Hence, PSC provides an elegant but computationally expensive method to solve crystal structures in a stepwise approach, splitting the full structure into one-dimensional projections.

In the present work, we develop further theoretical approaches to treat all aspects of *m*-dimensional PSs, in particular generally applicable linearization routines to parameterize the isosurfaces for efficient functional handling as well as computational storage and determination of intersections. The PSC algorithms are enhanced to treat the artificial values of the atomic scattering factor of scatterers in the *m*-dimensional PS. The implemented capability to overcome the previously employed equal point atom (EPA) model (see Section 2.2) improves PSC in the direction of a generally applicable structure determination approach. However, to test the developed algorithms and code, in this article, we determine the centrosymmetric structures only in two- and three-dimensional PS. Nevertheless, on the first estimation, the derived equations may be straightforwardly enhanced towards higher dimensions, which will be the focus of our continuous research efforts.

This study is structured as follows: The subsequent sections provide an in-depth exploration of our approach, commencing with the fundamental theory underlying the PSC-based framework. Next, the steps involved in solving a maximum of three parameters in the one-dimensional projection are discussed, including a general description of the linear approximation of isosurfaces. Finally, we present noteworthy generalizations based on Monte Carlo simulations for structure determination within the two- and three-dimensional centrosymmetric PS.

## 2. Theoretical methods

Solving a crystal structure is a meticulous process that involves the precise determination of the position of each atom within the crystal. In the context of PSC analysis, an effective strategy is employed, making use of a linear approximation of the isosurfaces defined by intensities derived from X-ray diffraction data within the corresponding PS. Careful analysis of the experimentally measured intensity of each reflection facilitates the reconstruction of the potential combinations of atomic coordinates by utilizing sophisticated piecewise analytic hyper-surfaces (Fischer *et al.*, 2005 ▸; Zschornak *et al.*, 2024 ▸).

### 2.1. Parameter space and isosurfaces

The complete solution of a structure with *m* atoms in the unit cell consists of 3*m* parameters, accounting for the three distinct coordinate components (*x*, *y* and *z*) of each atom. The 3*m*-dimensional space containing all possible combinations of those parameters is called the parameter space. By employing a one-dimensional projection of the crystal structure onto the main axes, the complex task of solving 3*m* coordinates is separated into several distinct steps, each solving *m* coordinates in the *m*-dimensional PS 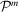. Then, assuming oblique reflections (more than one non-zero component), the relationship between the *x*, *y* and *z* components of atomic coordinates can be assigned (Fischer *et al.*, 2008 ▸). However, the necessary steps to combine the projections into a complete structure solution will be the topic of a further article.

Here, we adopt the projection onto the *z* axis as a representative projection for all axes, while employing the Miller index *l* to abbreviate the relevant X-ray reflections 00*l*. In general, the PS 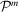 consists of *m* orthonormal axes, which we assign to the *z* components 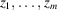 of the atomic coordinates. When considering a specific reflection, each point 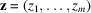 in 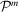 has a uniquely defined amplitude, which is directly related to the intensity (see Section 2.2). However, a specific amplitude value can be achieved through several 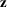, resulting in an ‘isosurface’. The isosurface embodies the entirety of possible combinations of atomic coordinates that generate the same amplitude for a specific reflection within the intricate landscape of 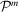. Each reflection provides insights into the possible configurations of the crystal structure: only the well defined combination of atomic coordinates described by all the isosurfaces can create the experimentally determined amplitude. The precise structure solution is, thus, defined as the intersection of the isosurfaces corresponding to different reflections [see Fig. 1 ▸(*d*)]. Through detailed and precise analysis, we unravel the regions where these isosurfaces intersect, which are ultimately interpreted as the coordinates of the atoms within the crystal structure. For error-free intensity values and using the isosurfaces directly, only *m* different reflections are required to solve for *m* coordinates, realized as the intersection of *m* isosurfaces. However, in the cases of linearized isosurfaces (see Section 2.4) as well as experimental and thus naturally erroneous values, the isosurfaces gain volume, and, therefore, so does the intersection region. An exact point solution cannot be determined; however, adding more reflections will improve the accuracy of the solution.

### 2.2. The structure factor

The basic requirement of structure determination is information about the Miller index and its corresponding intensity *I*, which can be expressed as 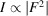, with the structure factor *F*. The complete structure factor is expressed as a weighted sum over atomic scattering amplitudes 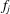 of all atoms present in the crystallographic unit cell. However, the natural disorder due to lattice vibrations and defects consequently leads to averaging over all unit cells using the parameters site occupancy 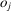 and Debye–Waller (DW) factor 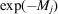. The DW factor accounts for the reduction in scattering amplitude caused by the positional uncertainty of atom *j* at 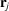, leading to the expression (Richter *et al.*, 2018 ▸)
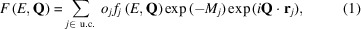
where u.c. is the unit cell. As a simple treatment, temperature-induced lattice vibrations are approximated in the harmonic limit by the mean square displacement 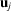 of atom *j* in the direction of the momentum transfer vector 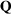 via 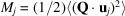. The anisotropic nature of atomic displacements is typically described using the anisotropic displacement parameter tensor 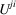, which provides a more comprehensive representation of the mean square displacement (Trueblood *et al.*, 1996 ▸). Nevertheless, as a first attempt to develop the linearization method, equation (1) is adapted by assuming that the atomic sites have full occupancy (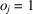) and are not influenced by temperature (*i.e.* the DW factor is neglected). These additional degrees of freedom can be easily implemented as additional dimensions within the PSC approach. They will specifically affect the topology and values of the respective isosurfaces. The precise application and validation are subjects for further developments of the code during the next phase of the PSC project. Also, we will constrain the discussion to centrosymmetric cases, which provides the advantage that the solution is not a complex value but lies in the real number space. Thus, we can express the structure factor, equation (1), as (Fischer *et al.*, 2005 ▸; Kirfel *et al.*, 2006 ▸)
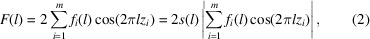
where *l* is the Miller index of reflection 00*l*, *m* is the number of atoms in the unit cell, 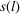 is the sign of the expression, 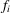 is the atomic structure factor (a real number, considering only Thomson scattering without resonance corrections) and 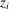 is the coordinate of the atom with index *i*.

If all atoms are considered to be equal (and point-like), then their scattering factors are identical, 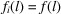, and can be set to unity; hence, equation (2) becomes 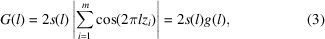
where 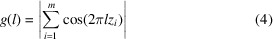
is the modulus of the geometric structure factor *G*. Detailed relationships for atoms on special positions are discussed by Fischer *et al.* (2005 ▸). The case of identical point-like atoms is referred to as the EPA model hereafter. The case of realistic scattering factors will be referred to as the non-EPA model.

Note the difference between the isosurface, *i.e.* the manifold, and the structure factor in terms of parameter dependencies. The isosurface is a subspace of dimension 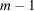 in 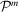, fulfilling a boundary condition of coordinates 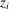 with a certain amplitude or intensity. For experimentally observed or theoretically calculated amplitudes 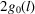 and 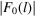, the isosurface of the geometric structure factor is mathematically expressed for the EPA case as
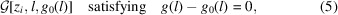
and that of the structure factor for the non-EPA case as
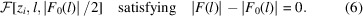
For better readability we use the expressions 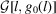 or 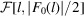 for EPA or non-EPA cases, respectively, where only *l* and 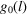 or 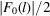 are specified explicitly. Further, the (geometric) structure factors 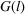 or 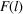 represent fixed structures 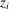 and only depend on *l*. Otherwise, simply 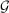 or 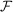 are used for EPA or non-EPA cases. The isosurface can be defined from given amplitudes 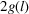, 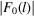 or intensities 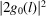, 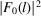, which is indicated with the respective index *A* or *I* on 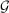 or 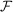.

The value of the amplitude 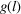 for a given reflection *l* is visualized as a function of the atomic coordinates 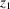 and 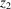 as color maps in Fig. 1 ▸. Also, Fig. 1 ▸ shows an isosurface corresponding to an arbitrary set of amplitudes 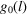, represented by black lines. To account for the complexities of realistic applications including different atoms and their scattering behavior, we will also consider various combinations of scattering factors. Although 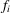 are complex quantities influenced by factors like the energy of the incoming X-rays and inter-lattice distances (Woolfson & Hai-Fu, 1995 ▸), we have carefully chosen appropriate real numbers representing heavy, medium and light atoms.

We realized that varying scattering factors 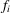 affect the topology of the isosurfaces [*cf.* equation (2)]. In EPA, 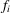 are set to unity, and hence the isosurfaces 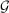 with large 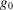 values are almost a circle in 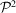 and a sphere in 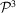 (*cf.* Figs. 1 ▸ and 2 ▸). In the non-EPA model, the isosurface 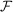 exhibits a variety of topologies, that require careful and independent handling (Section 2.4.1). Furthermore, the overall appearance of the isosurfaces in the PS is influenced not only by the ratios of scattering factors but also by the reflection index *l*. As the index *l* increases, the number of disjoint isosurface regions also increases (see Fig. 1 ▸). Section 2.4 gives more details on the systematic approaches.

The detailed analysis of intrinsic symmetries can contribute to a further reduction of the computational effort, by reducing the possible solution space 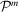 to the asymmetric PS 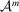. Those symmetries comprise (i) the centrosymmetry of the structure, (ii) the permutation symmetry for equal or partially equal atoms, and (iii) the choice of origin. The assumed centrosymmetry leads to the spatial limitation of the PS to the range 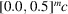, where *c* is the lattice constant of the crystal in the specified projection.

**Figure 2:**
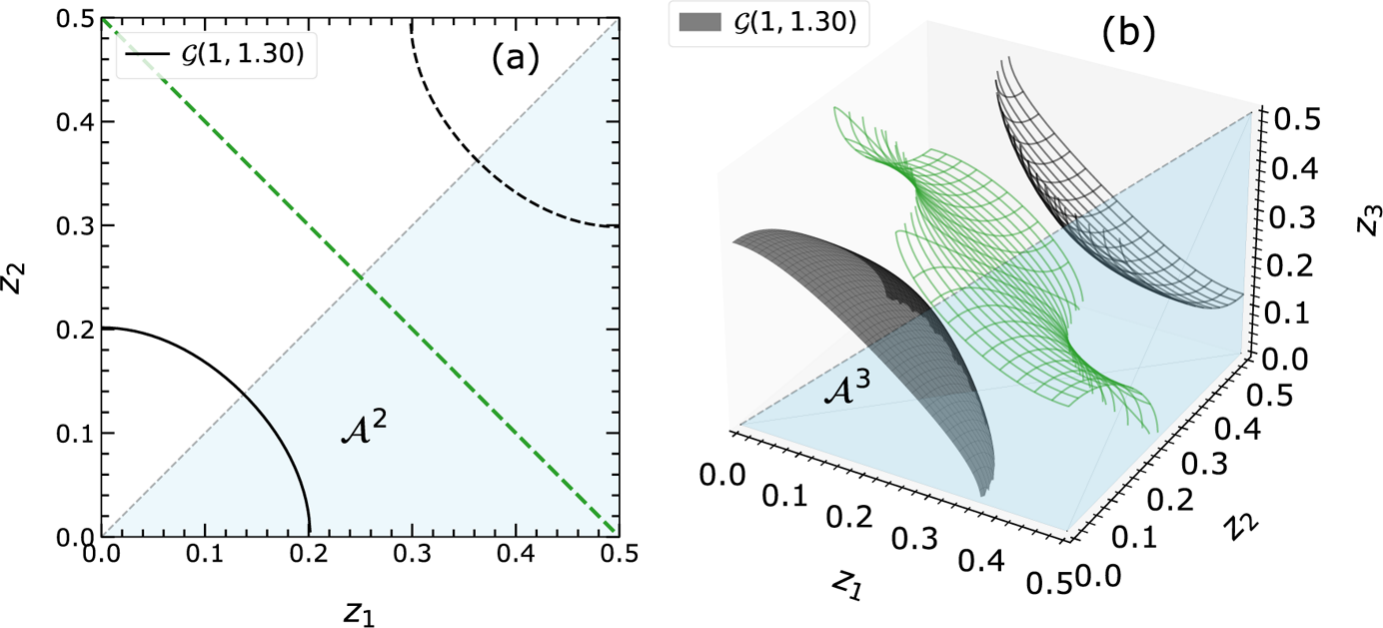
Isosurfaces 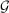 for reflection 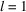 in two- and three-dimensional PS in (*a*) and (*b*), respectively, with highlighted asymmetric parts 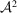 and 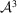. The black continuous line in (*a*) and solid surface in (*b*) represent the instance of the isosurface with the positive sign [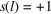]. The black dashed line in (*a*) and wireframe in (*b*) represent the negative instance [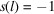]. The blue shaded regions represent 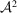 and 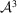 of 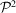 and 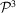, respectively, and contain the contributions from both 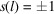. However, the knowledge or selection of 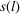 of the amplitude further restricts 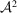 and 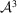, reducing the volume by a factor of two via the zero 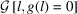 isosurface represented by the green dashed line in 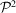 and wireframe in 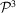.

**Figure 3:**
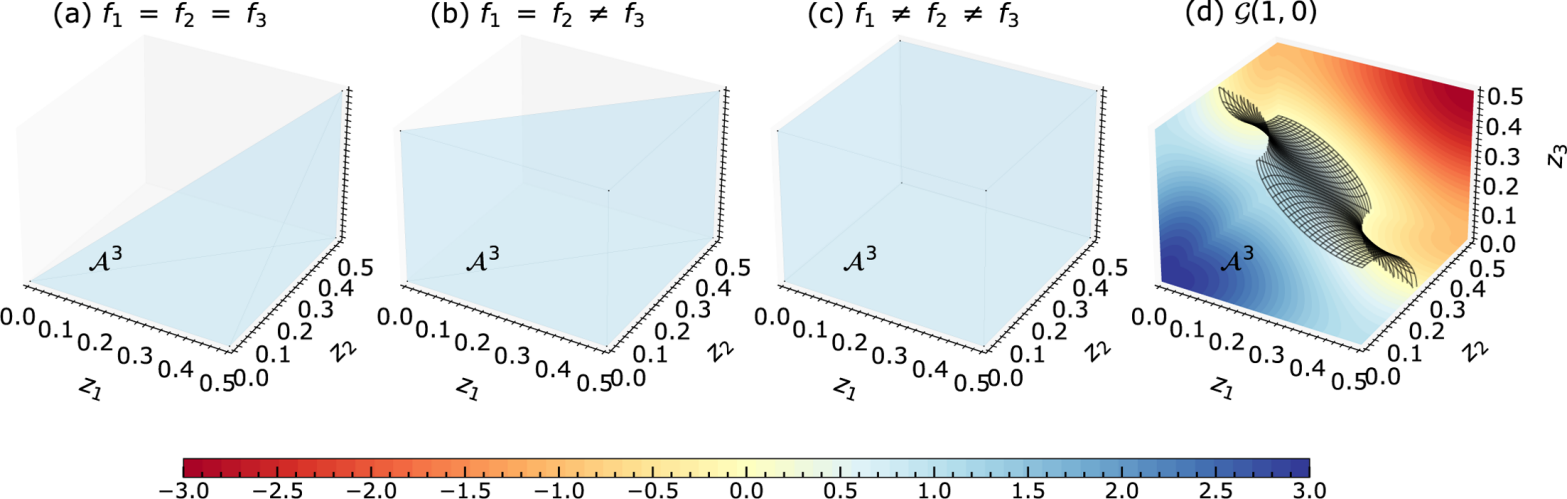
The definition of asymmetric units upon specific sets of *f* values is shown in (*a*)–(*c*). The 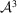 in (*a*) is defined for the EPA framework where all 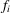 are equal. The 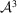 in (*b*) and (*c*) are defined for the non-EPA framework. In (*b*) it is assumed that two *f*s (along the 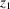 and 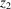 directions) are the same and 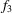 is different. In (*c*) it is assumed that all *f*s are different and hence 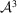 is equivalent to 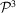. In (*d*) 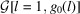 are given for the EPA case representing the magnitude of 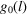 from 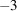 to 3 in color code. The zero isosurface 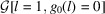 separates the PS into two halves containing isosurfaces 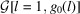 of positive or negative signs. When applying the choice of origin symmetry, the origin is fixed at 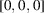. Then the asymmetric unit reduces to half under the zero isosurface, containing only the positive signs 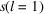, for the solution search. The color bar gives the magnitude of 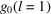 with the applied sign.

Furthermore, following the discussion by Fischer *et al.* (2005 ▸), in the case of EPA, the full PS 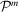 can be reduced by permutation of atomic coordinates, which encompasses both positive and negative instances of the isosurfaces. This reduction can be visualized using the corner points fixed at coordinates 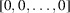, 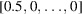,…, 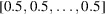 (*cf.* entire shaded area in Fig. 2 ▸). Even in the non-EPA cases, the occurrence of *n* equal atoms will induce limited permutation symmetry within the respective subspace of the PS, for which a reduction of 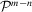 towards 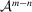 can be obtained. The possible cases for 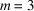 are shown in Fig. 3 ▸. As an example, if 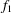 and 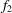 are equal, then 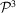 is halved and 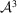 is a triangular prism as shown in Fig. 3 ▸(*b*).

In addition to the permutation of atomic coordinates, the choice of the origin of the crystal system at one of the two centers of inversion (at 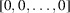 and 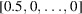) provides another intrinsic symmetry to reduce the possible solution space where the given structure resides. The composition of the set of 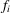 defines a specific zero isosurface, one boundary of the asymmetric unit given by the isosurface 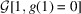 [*cf.* Fig. 3 ▸(*d*) as an example for 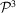]. Once the choice of origin is utilized only the blue region below or above the zero isosurfaces needs to be analyzed. These boundaries simultaneously define the linearization boundaries and the allowed solution space for the structure investigation.

Additionally, 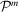 can be reduced using the sign of the isosurfaces if available; this is represented for 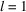 by the separation of the shaded region in Figs. 2 ▸(*a*) and 2 ▸(*b*) via the zero isosurfaces 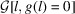.

### 2.3. General linearization approach within PSC

**Figure 4:**
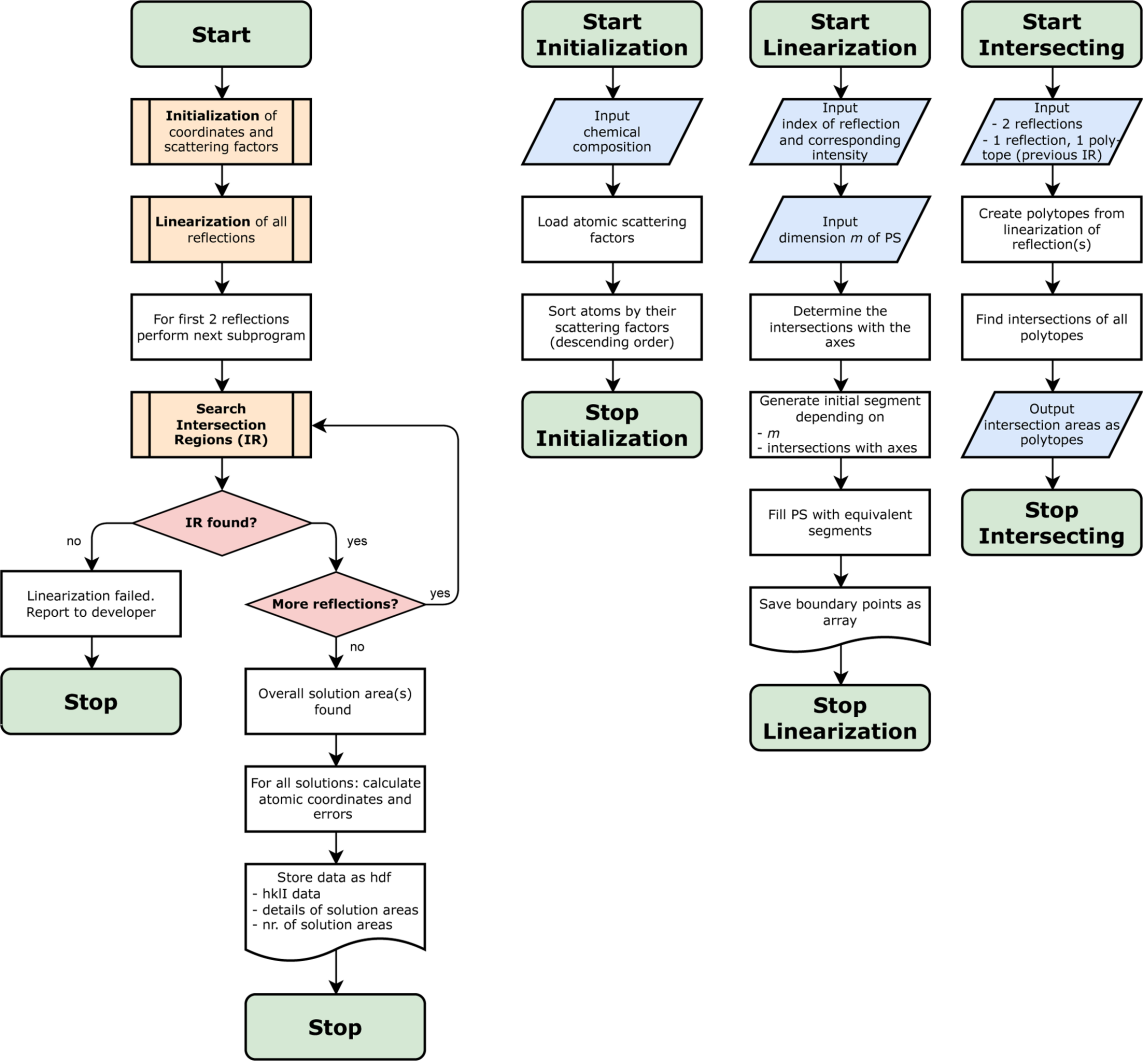
The scheme of operations flow in PSC includes the initialization, linearization and solution-finding process. Also, many intermediate decisions are made to verify the inclusion of all reflections, reaching intersection regions, imposing an accuracy limit on the area/volume of intersection regions *etc*. At the end of the structure determination, detailed outputs including reflections and their polytopes, intermediate intersection results, found solutions, the volume of each polytope, and the error on computed 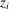 are written in HDF-formatted files.

The primary objective of structure determination in 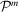 is to identify the intersection of isosurfaces 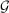 or 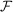 corresponding to different reflections (see Fig. 4 ▸). However, finding the intersection point directly is challenging as it involves complex trigonometric functions [*cf.* equation (2)]. One possible approach to reduce complexity, the focus of this article, consists of employing linearization techniques, which enable the replacement of intricate trigonometric expressions within linear boundaries. This approximation can simplify the problem and utilizes set theory to explore all potential solution regions. Note that the linear approximation may yield multiple solution regions as a result of step-wise intersections of linear approximants of different reflections. The approximation process generally involves the following steps, depicted in Fig. 4 ▸.

*Initialization*. In the first step, we implement specific measures to ensure the flawless execution of the linearization algorithm. This important step involves arranging the atomic structure factors 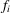 in a descending order, which helps us to determine the general curvature of the isosurface 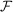, as well as to apply the permutation symmetry. The larger 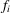 values correspond to heavier atoms and exert maximum control over the behavior of 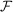, while the smaller 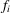 values pertain to lighter atoms and thus smaller contributions in the interference dependencies. The influence of 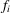 on the curvature of 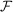 is depicted in Section 2.4.1. In this work, we distinguish between different types of topologies: they are called closed if 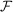 intersects all parameter axes and open if 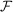 does not intersect with at least one axis.

*Linear approximation*. The simplest approximation method is linearization. We bound the curved isosurfaces of each reflection by two straight, parallel lines in 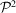 or two planes in 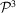. The normal vector and distance from the origin are the required descriptors of the boundaries and are being determined in this step. The equations and routines for these parameters are described in detail in Section 2.4. Notably, the linear approximation invokes the mean-value theorem at the core (Lozada-Cruz, 2020 ▸; Sahoo & Riedel, 1998 ▸). The challenge is the development of a new non-EPA linearization framework, which we present here for the first time, giving algorithms to linearize the isosurfaces governed by different atomic scattering factors. This non-EPA framework elevates the capability of PSC to handle realistic X-ray diffraction data.

*Solution finding*. Subsequently, the boundary descriptors obtained from the previous step are used to construct polytopes, described by a system of linear inequality equations with the number of variables identical to the dimension *m* of the PS. These polytopes are reflecting the PS region that matches the observed intensity for the specific reflection in linear approximation. The goal is to find the common regions that are enclosed by all polytopes of every reflection and that represent the projected atomic coordinates of the structure under investigation. To achieve this final solution, we search for the intersection regions of the polytopes created for successive reflections. In ideal cases, *i.e.* error-free amplitudes for a full set of reflections starting from 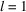, a single polytope solution region that uniquely represents the given structure may be identified. However, in many cases, PSC can yield multiple polytope solution regions in a consecutive intersection step for an arbitrary reflection *l*, or when taking into account intensities as observable without the knowledge of the structure factor’s phase.

*Data writing to HDF*. After the solution-finding process, we obtain crucial information such as the volume of solution regions, the coordinates of the edges of these regions, the number of solutions and the computation time. These data are stored in a file. Additionally, details about processed reflections, the atomic scattering factor, the structure factor, and experimental or theoretical intensity/amplitude are also saved for future reference. We have chosen to use the hierarchical data format (HDF) due to its versatility as a data model, making it ideal for managing large and complex data sets (The HDF Group & Koziol, 2020 ▸). HDF allows for the storage of various types of data within a single file. There is an efficient Python library available that facilitates the integration of this specific file format into our routines. This format guarantees that the data are easily accessible and portable across different platforms, thereby enabling seamless analysis and sharing of results, making it a superior choice for data storage and retrieval. Furthermore, data stored in HDF files can be easily visualized using the *HDFView* tool, enhancing the practicality of this choice (Group & Koziol, 2020 ▸). More details about *HDFView* are given in the supporting information (see Section S2).

### 2.4. Linear approximation routines

Solving for the intersection point 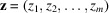 on a dense grid in 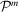 will become computationally expensive, challenging and sometimes even impossible for higher dimensions. However, we can reduce the computational effort significantly by solving for linearized approximations of the isosurfaces. Unfortunately, instead of solution points, linearization introduces expanded solution regions, which ideally should be kept as small as possible. Here, we apply a linear approximation as described by Fischer *et al.* (2005 ▸): we develop a basic unit called a ‘segment’ to linearize a well defined part of the isosurface and extend the linearization by shifting and rotating the segment to create a full cover-up of the isosurfaces within 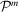. The segments consist of a set of parallel boundaries, the inner and outer limits of the isosurface with respect to the PS origin, and limiting boundaries perpendicular to each 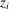 direction. We have discovered that, in addition to the circle-like topologies considered by Fischer *et al.* (Fig. 2 ▸), band-like isosurfaces can also occur. The origin of these and also the different handling within the linearization procedure will be discussed in Section 2.4.1.

**Figure 5:**
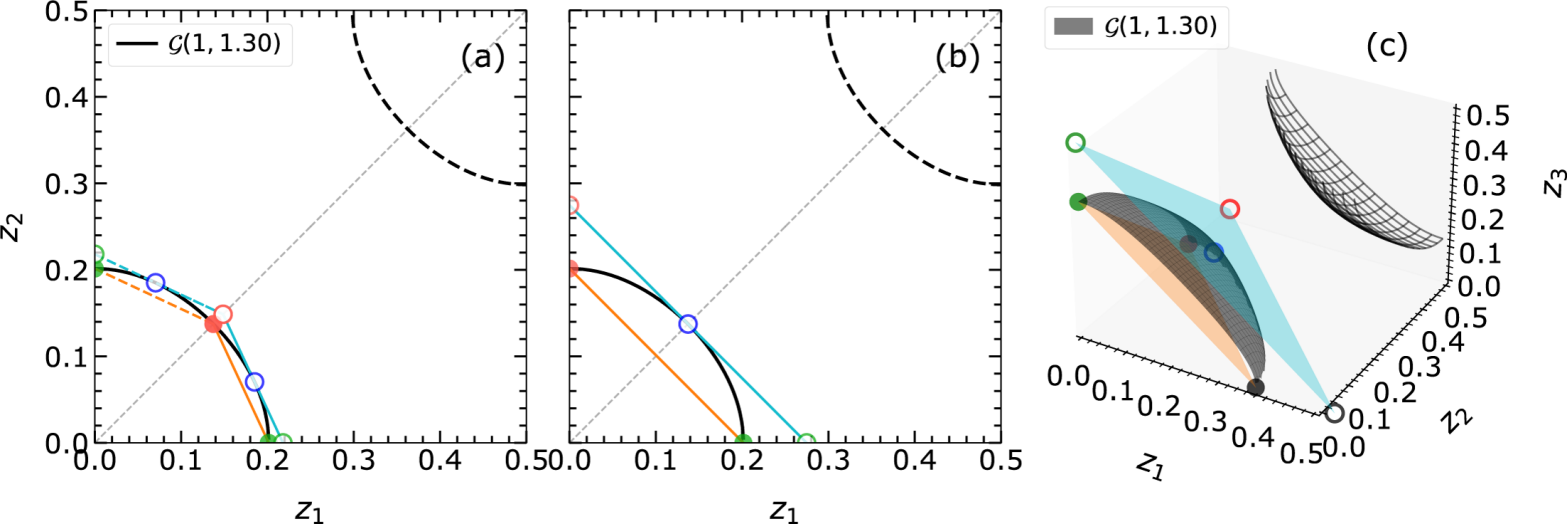
Linearization of an isosurface 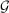 with (*a*) two segments in 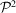, (*b*) one segment in 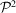 and (*c*) one segment in 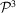. The filled and open circles represent the coordinates of the inner and outer boundary line/plane, respectively. The tangent point is represented by a blue open circle. The gray dashed line represents the mirror plane in 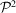 along the 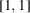 direction created by the EPA model. An arbitrary value of 1.3 for the magnitude 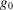 is selected and the isosurface is shown for reflection 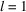 in 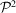 and 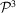.

Examples of the process of linearizing 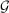 for the EPA case are shown in Fig. 5 ▸. The complete procedure to accomplish linearization is explained in the following sections; steps include the generation of segments around the origin of the PS as well as the repetition of segments in the PS.

#### 2.4.1. Topologies of isosurfaces

In previous literature, the isosurfaces were always described as circle- or sphere-like, resulting in closed loops in 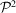 and 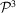. Fischer *et al.* have not introduced the concept of topology as it was not a requirement, since only the EPA models were applied, in which most of the hyper-surfaces are closed circle- or sphere-like. However, during our extensive investigations using Monte Carlo simulations (see Sections 3.2 and 3.3), we learned that this is not always true, and open, band-like structures can appear.

**Figure 6:**
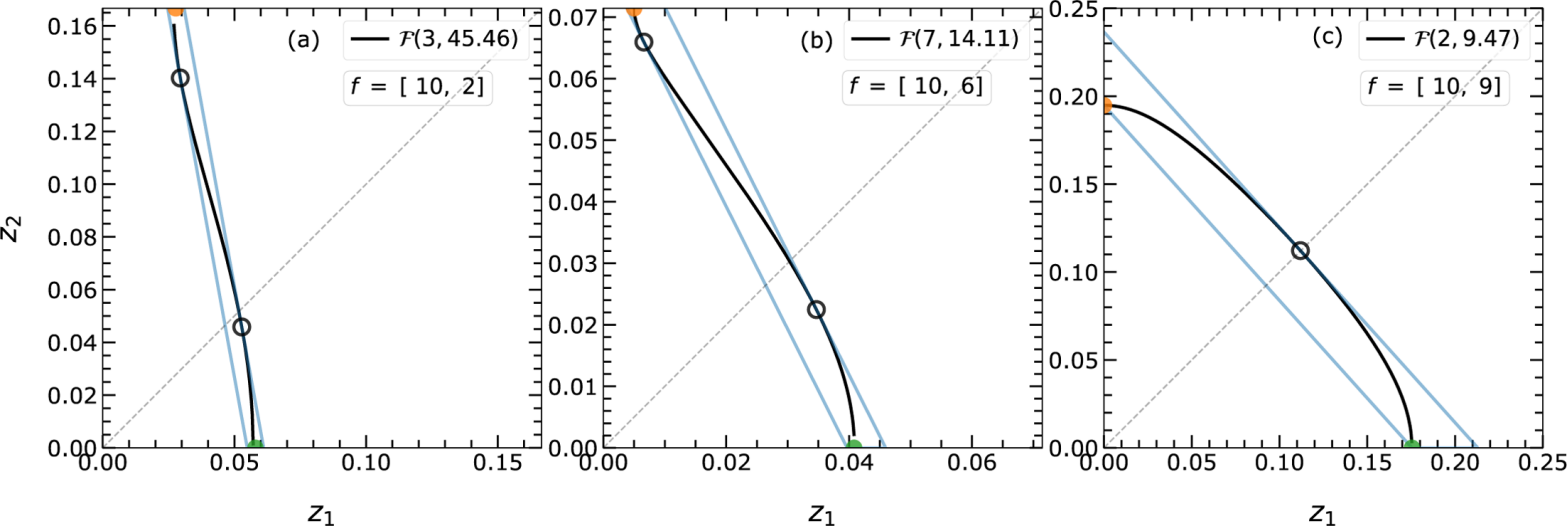
The different possible topologies of isosurface 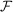 in 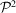 on varying atomic scattering factors 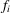 in equation (2). The 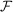 can be categorized according to their intersection along the 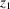 and 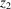 directions. The 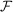 in (*a*) and (*b*) exclusively intersect the 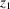 axis (open topology) and that in (*c*) intersects both the 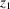 and 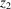 axes at different distances from the origin (closed topology). The developed algorithm handles and approximates these 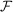
s alike. The boundaries from the approximation are shown by blue lines. The schematic demonstrates the effect of the ratio between 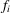 on the curvature of 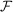. The very first segment of the linearization around the origin is shown and all repeated segments are avoided for better visibility. The filled green and orange points are used to get the slope and the found tangent points are shown by open black circles.

In general, the values of the atomic scattering factors are found to influence the curvatures of the isosurfaces. We learned that an anisotropic 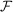 may show cases with an open topology along the direction where the less contributing 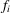, *i.e.* the smaller 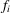, are assigned. For cases of similar atoms, where all structure factors 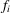 are alike (*e.g.* EPA model), closed topologies appear. Some of these isosurfaces have been examined and are presented in Figs. 6 ▸ and 7 ▸. The different shapes result from varying ratios of the atomic scattering factors.

A complete overview of the different isosurfaces 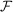 in 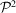 is realized via keeping 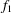 at a constant value of 10 and varying 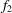. As 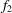 increases, the curvature of the isosurface changes, resulting in two categories of 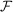: those that cut both main axes (closed topology) and those intersecting with only one main axis (open topology). Fig. 6 ▸ shows that 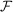 bends and tends to intersect both axes 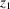 and 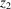 as 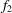 increases and becomes more similar to 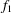.

**Figure 7:**
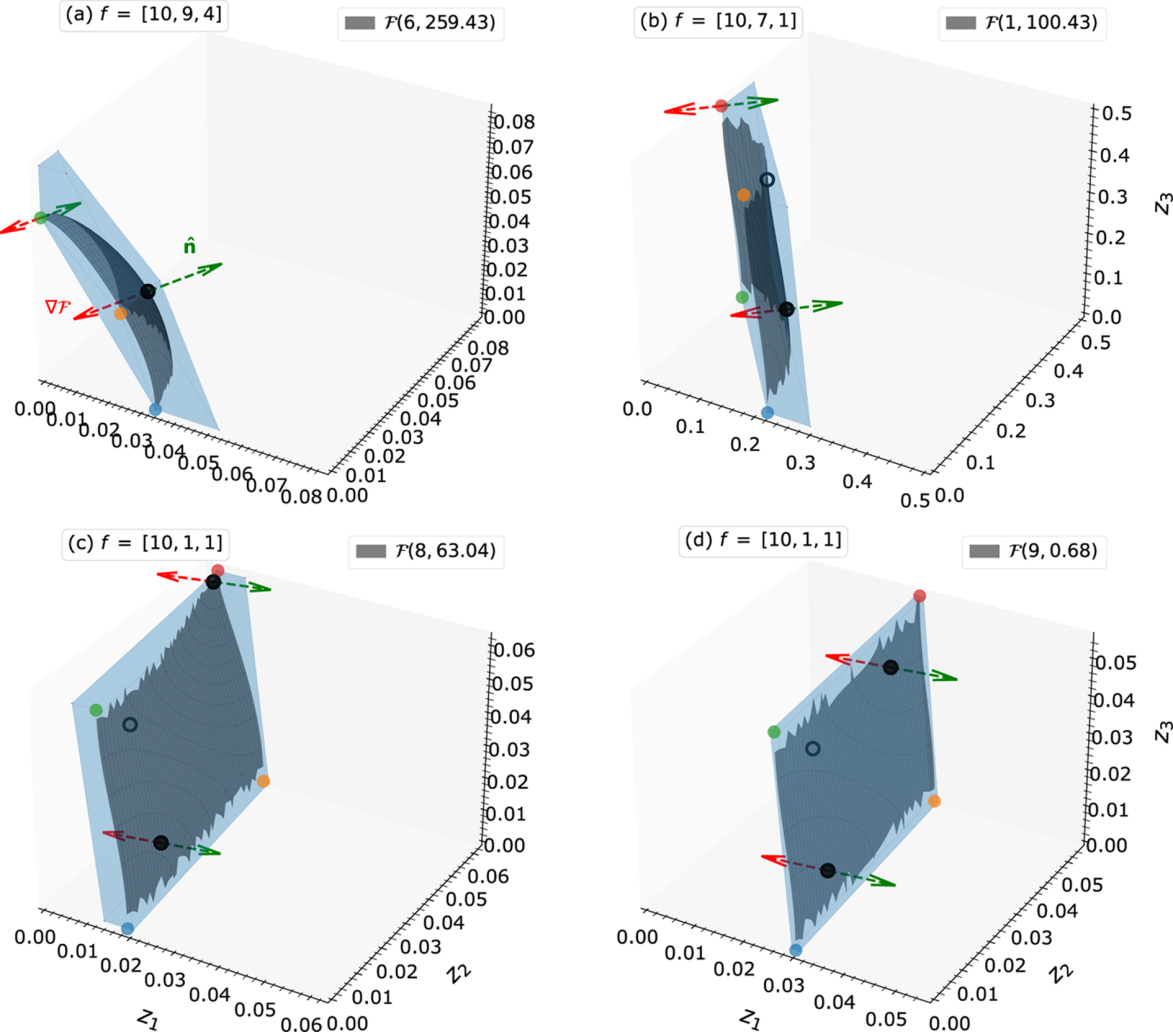
The different possible topologies of 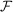 in 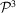 on varying atomic scattering factors 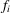 in equation (2). The 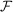
s are differentiated according to their intersection along the 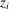 directions. They can intersect (*a*) with all three axes (closed topology), (*b*) only with the two axes 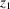 and 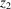, or (*c*) and (*d*) only with axis 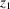 (open topologies). The very first segment of the linearization around the origin is shown and all repeated segments are avoided for better visibility. Also, the computed gradient of 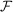 and the normal vector are depicted at different tangent points. The filled, colored dots are used to define the required normal vector. The tangent points representing planes nearest to and farthest from the origin are represented by solid black dots, while the open black circle indicates an additionally computed tangent point.

A similar study is carried out in 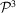 by assuming different 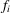 combinations. Fig. 7 ▸ summarizes the observed isosurfaces upon varying the ratios of the scattering factors 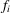. As in 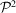, the isosurface in 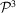 may exhibit an open topology along the 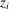 direction, which is associated with a low scattering factor 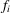 [*cf.* the open topology along 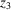 in Fig. 7 ▸(*b*), or along 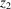 and 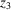 in Fig. 7 ▸(*d*)]. The isosurface intersects all axes when all 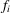 are similar.

In order to control the direction where the open topology may appear and to simplify the cases that may occur during the PSC approach, we sort the atoms according to their scattering strength in the initialization step such that the atom with the smallest scattering factor is associated with the highest index of *z*.

#### 2.4.2. Generating the segments

The linearization of any complete isosurface 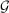 or 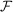 starts with defining a segment in the vicinity of the origin. The concept of linearization can be easily understood in 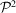 and can be systematically extended for higher dimensions. The isosurfaces for a given *l* have a period of 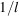, *i.e.* each isosurface is repeated in each complete PS in translations of 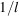 along each axis. Hence 1, 1/2 and 1/3 are the periods for *l* = 1, 2 and 3, respectively [*cf.* Figs. 1 ▸(*a*)–1 ▸(*c*)]. This characteristic period assists in the linearization.

The first task is to find the coordinates 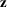, where the isosurface intersects with the axes in order to define the inner and outer boundaries. In 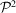, this coordinate can be determined by rearranging equation (2) to 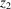, while 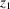 is set to zero: 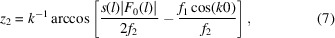
where 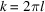 hereafter. In the case of 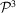, setting two of 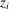 (for example 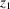 and 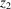) to zero yields the remaining 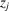 (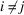), *e.g.*
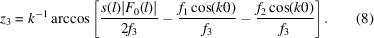
These intersections with the main axes are represented by the filled points in Figs. 5 ▸(*b*) and 5 ▸(*c*). The determination of the boundaries differs significantly for closed and open topologies (see Section 2.4.1). Therefore, we will discuss them separately.

*Closed topologies*. For closed topologies, all intersections with the axes exist and the inner boundary is devised by forming a line (in 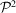) or a plane (in 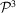) from those determined intersection points. The filled points in Fig. 5 ▸ define the inner boundary described by a unique normal vector 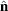, which assists in determining the outer boundary.

The outer boundary is defined by employing a parallel shift of the inner boundary. Therefore it is crucial to determine the corresponding tangent point (open blue circles in Fig. 5 ▸) on the isosurface with respect to the normal of the inner boundary. The line or plane formed at this juncture delineates the outer parallel boundary, marking the point beyond which 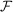 ceases to exist. The existence of this specific tangent point is assured by the mean-value theorem (Hobson, 1909 ▸), which – for the general non-EPA case – states that a normalized gradient of the isosurface equivalent to the unit normal exists. With knowledge of 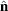 from the inner boundary, a tangent point can be found by solving 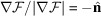; for derivations see Section 2.4.3.

*Open topologies*. For a given set of atoms with varying scattering contributions 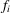 and a specific intensity or amplitude, the isosurfaces may show an open topology and not intersect with all the axes (see Section 2.4.1). In such cases, the curvature of the isosurface is not convex over the full period but switches from convex to concave in the local vicinity of the open topology. In consequence, lines and planes derived from the intersections (closed circles in Fig. 5 ▸) will cut 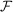. Thus, for these cases, the determined lines/planes must be shifted both towards the origin and away from it to define limiting but not restricting boundaries. Appropriate anchor points for these shifts are defined with a strategy similar to that used for the closed topologies: we search for the tangent points at the isosurface with respect to the normal vector of the line/plane defined by the intersection points. Due to the change between concave and convex behavior, at least two such tangent points exist, which serve as anchor points for inner and outer boundaries, respectively.

*Finalizing the segment creation*. Once the inner and outer boundaries are defined, the segment can be created using the boundary distances between the origin and the inner and outer boundaries. For a given *l*, the boundary distances and 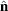 are unique and together they provide segment descriptors. This segment linearizes only a part of 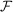 in the vicinity of the origin (*cf.* Section 2.4.1). The remaining parts of 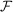 are linearized by utilizing the translational and rotational symmetries as described in Section 2.4.5. The routines are generally applicable, including the EPA case with geometric structure factor 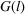 and the corresponding isosurfaces 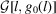.

#### 2.4.3. Finding tangent points

In the linear approximation, finding the tangent point is vital as well as critical. To find the tangent point, the least-squares (LS) refinement (Dekking *et al.*, 2005 ▸; Lawson & Hanson, 1974 ▸) is employed for the isosurface 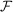 [equation (5)]. Within the refinement, the deviation between a desired normal unit vector 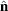 and the direction of the gradient of 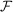 is minimized to identify the parallel hyperplane tangent to 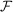. Illustrated for 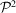, a selected initial point is shifted along 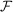 to meet the requirement
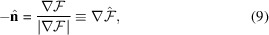
where 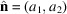 has the fixed components 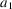 and 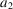 along the 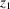 and 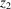 axes, respectively. At the required tangent point, the conditions 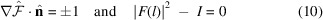
must be satisfied, with the sign 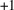 and 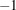 of 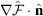 denoting the convex and concave curvature of 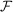, respectively. These conditions define the error function to be solved numerically. The points 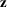 where 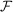 cuts the axes [calculated from equation (8), *cf.* the colored points in Fig. 7 ▸] and the point 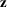 along the main diagonal {*i.e.* the 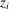 values of 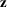 are equal, determined from 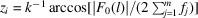 with 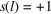} are considered as the different initial guesses for the possible tangent points to start the iterative process. Depending on the curvature of 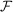, each initial guess can yield similar or different tangent points, which are dealt with differently for open and closed topologies.

The isosurfaces with closed topologies have a single convex curvature and will have a single tangent point [*cf.* Fig. 7 ▸(*a*)]. Isosurfaces with open topologies may also show additional tangent points due to concave–convex curvature change [*cf.* Figs. 7 ▸(*b*)–7 ▸(*d*)]. These tangent points as well as the intersection points of 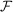 with the axes are used to determine the distance of the outer and inner boundaries. For the assumed case of 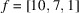, the single open topology results in two different tangent points [*cf.* Fig. 7 ▸(*b*)]. The tangent point closer to the origin gives the inner boundary distance, and the point further away is used to fix the outer boundary. The isosurfaces with double open topology result in more than two tangent points due to further changes in the curvature near all intersecting points [*cf.* Figs. 7 ▸(*c*) and 7 ▸(*d*)].

The above formulations are followed for a PS of dimension 3 or higher. However, the parallel boundary lines in 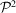 offer an alternative elegant solution to solve for the tangent point. The normal vector 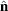 in 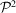 can be replaced by the slope ζ of the inner (and outer) boundary, which is defined as 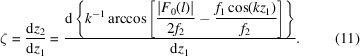 Using the identity 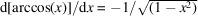 and for simplicity applying 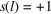, the above equation becomes 
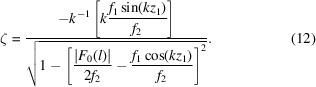
After resolving the root and rearranging the above equation, we obtain 
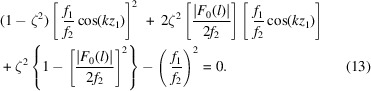
With 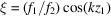 the above equation becomes
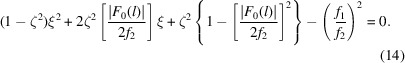
Solving this equation for ξ and 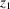 on the basis of the identified slope ζ from the inner boundary gives the linear function for the outer boundary as well. In particular, the two roots of equation (14) can probe all possible maxima or minima of 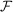. If both roots are valid, *i.e.* real and smaller than 1, then 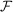 will have two tangent points [*cf.* Figs. 6 ▸(*a*)–6 ▸(*b*)], defining at the same time inner and outer boundaries. If only one root is valid or the two roots are identical, then 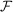 will have merely one tangent point defining the outer boundary [*cf.* Fig. 6 ▸(*c*)].

#### 2.4.4. Precision of the tangent point

Irrespective of topology, the exact linearization of the isosurfaces is important to reduce false structure predictions or the number of pseudo-solutions. The quality of the linearization of 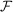, particularly in higher dimensions 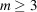, can be inferred from the metrics: (*a*) the intensity at the tangent point and (*b*) the angle between the normal 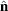 and the gradient 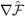 of the isosurface. The found tangent point must be on the isosurface and hence must result in the same intensity value as that of 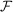. The incorrect prediction of the tangent point may lead to differences between the structure-immanent solution space and the approximation, which may, on the one hand, unnecessarily increase the volume of the linearization segment and, on the other hand, even more severe, exclude valid solution volume. The angle between 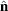 and 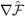 should be 180°, due to the anti-parallel condition of equation (9) and we monitor the discrepancy by the deviation angle. However, inherited from the mixed concave–convex curvatures of isosurfaces with an open topology, the intersecting points of 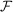 on the main axes are no longer co-planar, introducing the need for approximation on defining 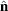. We apply the singular value decomposition (SVD) method (Campbell *et al.*, 2021 ▸) to determine the initial plane with the respective 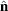 from the corner points [*e.g.* the blue, green, red and orange colored points in Figs. 7 ▸(*b*)–7 ▸(*d*)]. The found 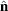 from SVD defines again the tangent point, the open black points in Fig. 7 ▸.

In Table 1 ▸, both intensity and deviation angle are given to estimate the quality of linearization of respective isosurfaces for the challenging cases shown in Fig. 7 ▸. For the given examples, the theoretical intensity (from the atomic structure) is identical to the one calculated from the tangent point; additionally the deviation angle is on the order of 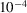 in all cases.

The current implementation still offers significant potential for enhancing the linearization method, specifically in resolving both inner and outer tangent points for cases involving open topologies and mixed concave–convex curvatures in higher-dimensional PS. One such enhancement could involve the development of a double-segment approach tailored for higher dimensions. Additionally, it is crucial that the determined boundaries fully encapsulate the entirety of the isosurface. To mitigate the risk of overlooking any valid solution spaces, we have introduced a cross-checking routine based on the grid-based method that verifies the complete enclosure of 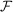 by the polytope. In the fail-safe case, the portion of the uncovered isosurface is returned to the momentary solution space, and the outer boundary distance is recalibrated, allowing for the continuation of the linearization process. However, this grid-based approach may demand substantial computational time, especially in higher-dimensional PS. Therefore, the development of a more efficient algorithm that guarantees the complete enclosure of 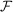 while also validating the accuracy of the determined normal vectors remains a priority for future research and development efforts.

#### 2.4.5. Completing the linearization

The segment obtained in the previous step only linearizes a part of the isosurfaces with 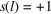. The complete 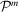 encompasses 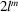 copies of this unique segment, due to *l*-fold mirror symmetry along each 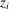 direction: *l* copies in all *m* directions, maintaining a modulo of 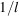 [see Figs. 1 ▸(*a*)–1 ▸(*c*)]. Before performing this translational repetition in PS, the formed first segment is rotated around and mirrored about the origin to enclose further parts of the isosurface 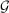 or 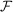.

**Figure 8:**
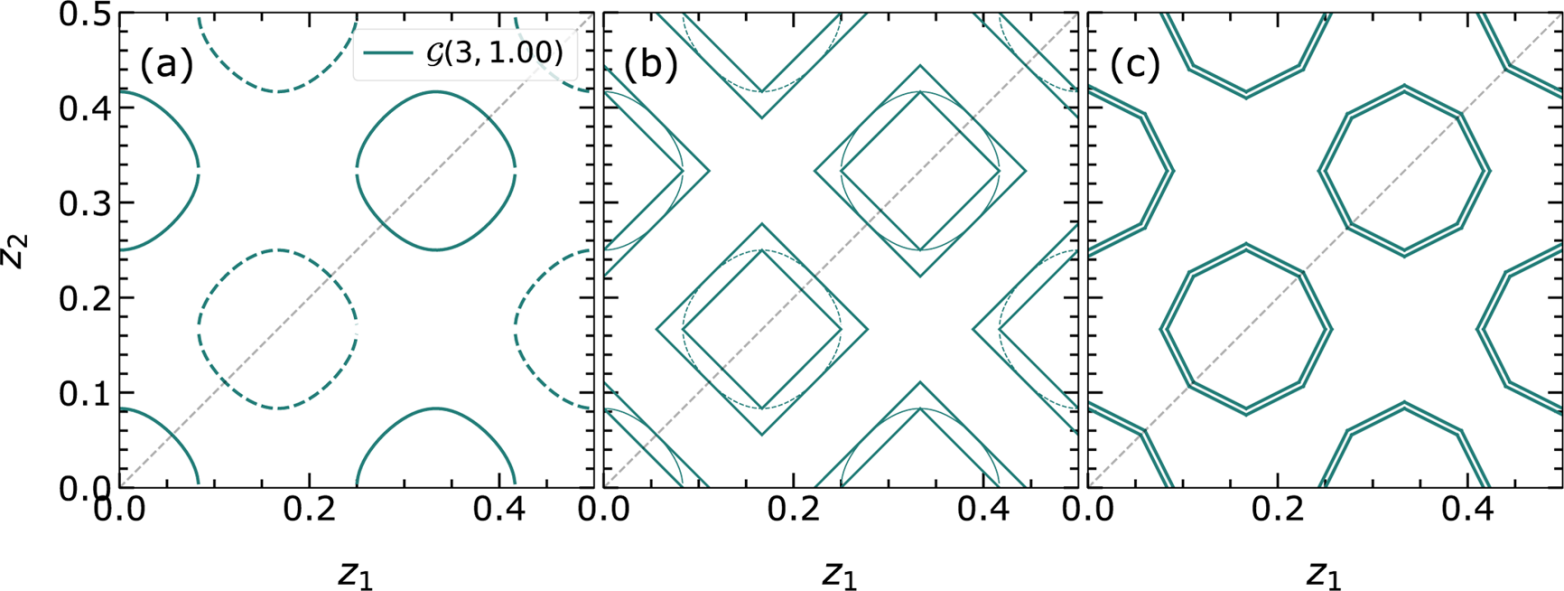
An example 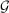 generated for 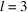 with 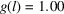 in complete 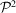. (*a*) 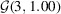 and their linearization (*b*) with single-segment and (*c*) with double-segment approaches. The continuous and dashed lines in (*a*) represent 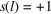 and 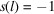, respectively. They are centered around the points with 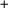 or 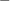 maximum amplitude.

The replication of the segment in our code is carried out using the mirror planes that pass through the origin. In 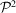 there are two different mirror planes represented using the vectors 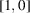 and 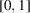 for 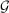 and 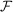 and two additional mirrors perpendicular to the main and secondary diagonals exclusively for 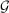. The mirror planes represented by the vectors 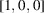, 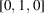 and 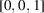 are used for 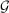 and 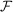, as well as additional mirrors perpendicular to the main and secondary diagonal exclusively for 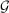. The segment descriptors are then multiplied by these vectors to form further rotated segments in both closed and open topologies (*cf.* Fig. 8 ▸ as an example for four/eight segments in 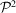). This initial set of equivalent segments is then repeated in PS with translation vectors 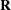 as follows: 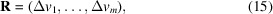
where 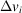 are the translations along the 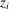 direction in 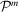, varying between 0 and 0.5 in increments of 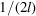. Those translations are applied only if the centers of polarity condition at maximum amplitude is fulfilled, demanding that all signs of the individual contributions are equal. The centers of polarity condition is defined as 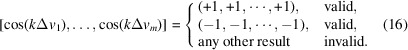
A 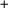 (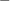) center is found if all elements in equation (16) are positive (negative). Any other combination is denoted as mixed translation centers and describes positions where there is no presence of maximum or minimum amplitude (*cf.* Fig. 8 ▸). For example, the combination 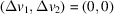 in 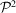
has 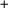 polarity, since 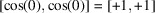. The combination 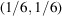 has 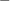 polarity because 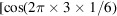, 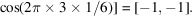
However, combinations such as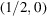 have mixed polarity, *i.e.*
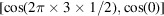
 = [−1, 1], and are invalid translation centers in 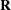 construction. Once the reflection *l* is defined, we can derive the translation vectors 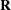 to ensure proper selection of 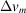 values. These 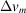 values are then utilized to replicate the segments and enclose the 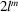 copies of 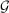 or 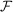 unique segments within 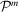. This iterative procedure effectively completes the linearization process.

#### 2.4.6. Improving the linearization

The explained procedure of linearization for 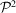 and 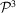 involves the definition of a single segment that fully encompasses the isosurface in the vicinity of the origin. However, the defined segment includes a rather large solution space and, thus, has the potential not only to minimize the unwanted solution space but also to suggest a new linearization technique. This can be achieved by increasing the number of used segments. We will refer to this approach as the double-segment linear approximation. Figs. 5 ▸(*a*) and 5 ▸(*b*) depict a comparison of these different linearization variations in 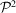 for closed topologies.

**Figure 9:**
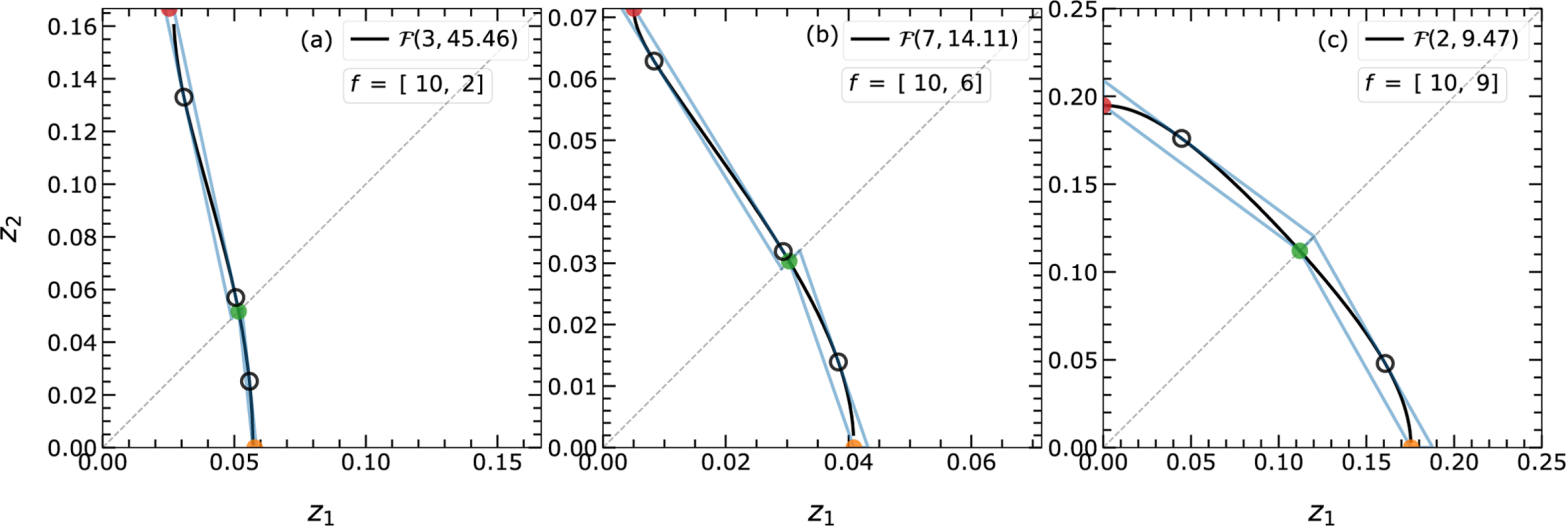
Application of the double-segment approach to the isosurfaces from Fig. 6 ▸. The filled green–orange (green–red) points are used to calculate the slope for the part of 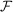 below (above) the diagonal line. The found tangent points are shown by open black circles. When the part of 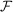 has a concave–convex mixed curvature, it possesses multiple tangent points for a given slope [*cf.* (*a*) and (*b*)] and equation (14) is capable of exploring all points in a single analysis. In the case of multiple tangent points, all points are used to define inner and outer boundaries.

The transition from the single- to the double-segment approximation is achieved by dividing the PS into two parts in the most advantageous way: at the main diagonal, where the components of 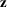 are identical. The intersection of the isosurface with the main diagonal, which is represented by the filled orange point in Fig. 5 ▸(*a*), *i.e.*
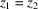
 = 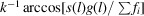, serves as the first point to determine the inner boundaries of the two desired segments. Then setting 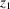 or 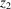 to zero results in the respective 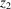 or 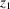 component of the second point [filled green points in Fig. 5 ▸(*a*)]. The two straight lines formed from these two points with the orange point define the inner and outer boundaries, similarly to the single-segment approach. Following the same methodology, the double-segment approximation is successfully developed also for non-EPA, only in 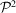 PS. As depicted in Fig. 9 ▸, the isosurfaces from Fig. 6 ▸ are approximated using double segments.

This improvement in linearization results in a significantly reduced solution space at the cost of additional computational load for handling a larger number of polytopes (*cf*. Sections 3.2.1 and 3.4.1). However, the variation in the approximation is exclusively developed for 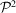 at the moment, while the single-segment linearization is employed for three- or higher-dimensional PS by default. Developing optimized variations other than single-segment linearization in higher-dimensional PS is an important future goal and will be considered in future work.

### 2.5. Intersection of linearized isosurfaces: the solution

Once the linearization is completed, the polytopes are created by collecting all segments for a given reflection to search for the solution region. The set of polytopes contains 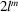 segments for a given reflection *l*. In principle, exactly one of the manifolds of an isosurface of reflection *l* always contains the crystal’s structure solution, and therefore the corresponding segments also do. In turn, we need to find that region that is common to all the isosurfaces of different reflections; this process corresponds to determining the intersection between the polytopes.

The solution is found systematically through the following procedure. The solution space is reduced by intersecting the polytopes of the different reflections. Hence, with the addition of more reflections to the calculation, the solution space and the positional errors are in general gradually decreasing. This process is repeated until the last available reflection. Often, the final solution region consists of several very small, disjoint areas that form clusters. Subsequently, the final solution regions are used to calculate the coordinates of atoms in the structure of interest; these coordinates are presented as a list of atomic positions with their errors, as usual in crystallographic information file format. To determine the atomic position and the error in the analysis, we utilize the centroid of the solution regions and the extension in the 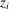 directions. The extension of each component can be quantified as 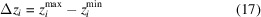
using the minimum 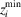 and maximum 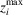 values. These set operations are carried out by means of the *polytope* (Filippidis *et al.*, 2016 ▸) and *shapely* packages (Gillies *et al.*, 2022 ▸). The polytopes shown in all figures are generated with the *IntvalPy* package (Androsov & Shary, 2022 ▸).

## 3. Results

In the upcoming sections, we present an introductory example (Section 3.1) and the results of Monte Carlo (MC) simulations of PSC to scrutinize the theory’s performance through a large number of simulations in 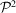 (Section 3.2) and 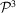 (Section 3.3). The examples will cover EPA as well as non-EPA calculations as the variation of the structure factor can have a severe impact on the shape of the isosurface and thus on the linearization process and the validity of a determined solution concerning real data.

The MC simulations are analyzed to gain insights into the overall solution landscape. The volume of the solution regions and the error are monitored in each addition of reflections to pinpoint any artifacts. For the given coordinate, we calculate and sum the volumes of all obtained solution regions as simple performance descriptors. This cumulative volume is then used to construct a virtual, representative, *m*-dimensional sphere. Ideally, we receive a singular final solution identified within the overall solution landscape. However, in the case of numerous solutions, all are equally probable within the limits of linearized polytope regions, highlighting the significance of considering all possible solutions in the analysis.

### Explanatory example in 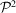


3.1.

**Figure 10:**
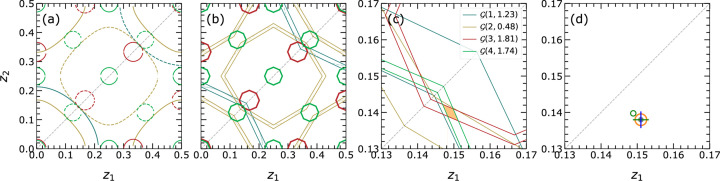
As a test case the coordinates 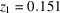 and 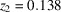 are solved by double-segment linear approximation and using the first four reflections for the EPA case and intensities with (*a*) the 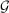 for 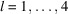, (*b*) the boundaries of linearization repeated in complete 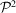, and (*c*) the common intersection region from the linearized polytope with a total area of 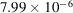. The dashed gray lines denote the mirror symmetry in 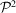 for EPA cases. The green open circle in (*d*) denotes the centroid of the solution region [the shaded area shown in (*c*)] which is predicted to be 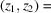

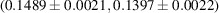
. The green and blue bars represent the calculated errors in 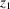 and 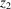 and are attached to the test structure’s assumed coordinates for reasons of comparability. A radius corresponding to the polytope area is calculated and represented as an open orange circle in (*d*).

The effectiveness of the linear approximation is evaluated by solving an example structure consisting of two atoms, with coordinates 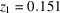 and 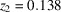. The structure is solved by considering the reflections 1 to 4 whose intensity is converted into the associated 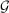 within the EPA framework, *i.e.* the scattering factor of the two atoms is set to 1. Figs. 10 ▸(*a*) and 10 ▸(*b*) illustrate the superimposition of all 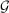 and their linear approximations, respectively.

The obtained isosurfaces 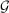 exhibit a predominantly smooth curvature and have a nearly circular topology in 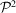 [Fig. 10 ▸(*a*)]. The behavior is trivial due to the application of the EPA model for intensities. The determined solution in 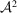 is 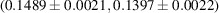, shown in Fig. 10 ▸(*c*). The error is on the order of 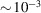, which can be reduced further by increasing the number of reflections (for example, *cf.* Section 3.2.1). The solution is unique in 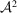 [as shown in Fig. 10 ▸(*c*)]; however, the mirror symmetries along the main diagonal lead to three additional, but equivalent, solution regions in 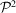.

If experimental errors are considered, they enlarge the width of the linearization polytopes and further distinct solution regions may appear (Zschornak *et al.*, 2024 ▸). The results are summarized in Fig. 10 ▸(*d*) with the centroid (green circle), area (orange circle) and extent of the solution region (cross). Including more reflections in the calculation may merge the green and orange circles, reducing error and further decreasing the area of the solution region. The schematic as in Fig. 10 ▸(*d*) is used to outline the results from the MC simulation in the sections below.

### MC simulations in 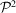


3.2.

The performance of the developed linearization technique and the accuracy of the PSC approach are analyzed through MC simulations. Within all PSC routine variations, the same set of random atomic coordinates has been used to ensure comparability. The trend of area or volume (in 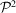 or 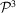, respectively) and extension of the solution region is monitored to track the deviations in structure prediction and identify weaknesses of the routines. Finally, the computing time is measured and presented for each MC simulation. The results are presented in the same style as in Fig. 10 ▸(*d*) with an additional open yellow circle highlighting the total number of solutions. This yellow circle is centered in the same way as the orange one and its thickness changes according to the number of identified total solutions. Hence, the thicker the yellow circles, the greater the number of solutions obtained. Also, timings and code performance for the MC simulations are evaluated in detail in Section 3.4.1.

#### 3.2.1. MC simulation within the EPA framework

At first, randomly generated coordinates are solved using the EPA model, *i.e.* the atomic scattering factors 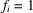. Since the sign and magnitude of the amplitude 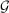 are calculated explicitly, the given atomic structures can be solved using the following two different approaches. For the amplitude approach 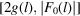, both sign and magnitude are utilized, and only the polytopes corresponding to the correct sign are considered in the calculation. In contrast, for the intensity approach 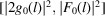, all polytopes are considered without knowledge of the sign. The amplitude approach is particularly beneficial when the experimental data contain the sign.

**Figure 11:**
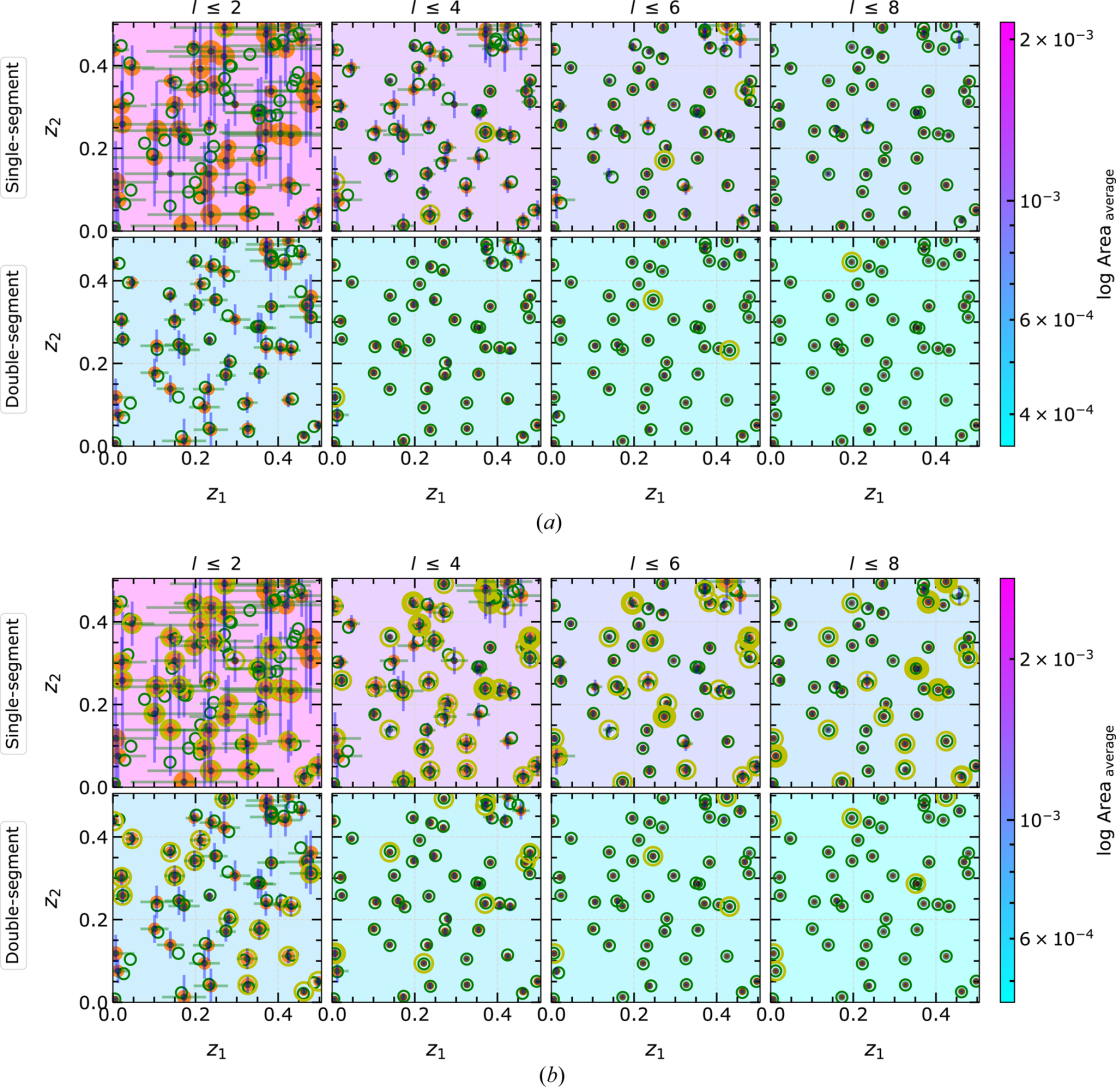
Results of MC simulation using single- and double-segment linearization with amplitude (*a*) and intensity (*b*) approaches. The green circles surround the centroid of the solution region within the EPA framework. When more than one solution region is available, the number is represented by the yellow circle (increased thickness for a larger number, *cf.* Fig. 15). The green and blue bars are the positional uncertainties on the computed 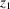 and 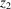, respectively. The area of all solution regions is summed to calculate the radius of the virtual circle as 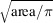, shown by the orange circle. The background color is defined by the average area of the solution regions of all random pairs, normalized to the overall minimum and maximum for all presented settings.

Figs. 11 ▸(*a*) and 11 ▸(*b*) illustrate the progression of the intersection region using both single- and double-segment approaches. As more reflections are added incrementally the positional uncertainties and the area of the solution region are continuously decreased. Fig. 11 ▸ shows that the double-segment linearization reduces the error more than the single-segment linearization. The double-segment approach provides higher resolution by a factor of 2.7 with just the first two reflections and 3.6 with eight reflections. The main reason is that the polytopes generated in the single-segment approach include a larger area; thus the intersection regions tend to remain larger than those from the double-segment approach. Consequently, the positional uncertainties on the computed 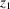 and 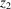 are higher in single segments. However, the area and positional uncertainties of the solution region exhibit steady improvement as more reflections are considered. Remarkably, with only four reflections, the double-segment linearization reduces the average area of the solution region below 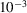, while the single-segment linearization achieves a comparable result after considering as many as eight reflections. These findings highlight the advantages of employing multiple segments in enhancing the accuracy and precision of the computed atomic coordinates.

The amplitude and intensity approaches solve the coordinates, resulting in a similar real solution. In most cases, the amplitude approach results in a unique solution in 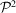. However, the intensity approach produces many equivalent mirror solutions due to ambiguity in the sign [*cf.* the difference in thickness of the yellow circles between Figs. 11 ▸(*a*) and 11 ▸(*b*)]. Hence, as known from conventional X-ray diffraction refinements, it is beneficial to measure both magnitude and sign for a unique structure prediction.

#### 3.2.2. MC simulation within the non-EPA framework

**Figure 12:**
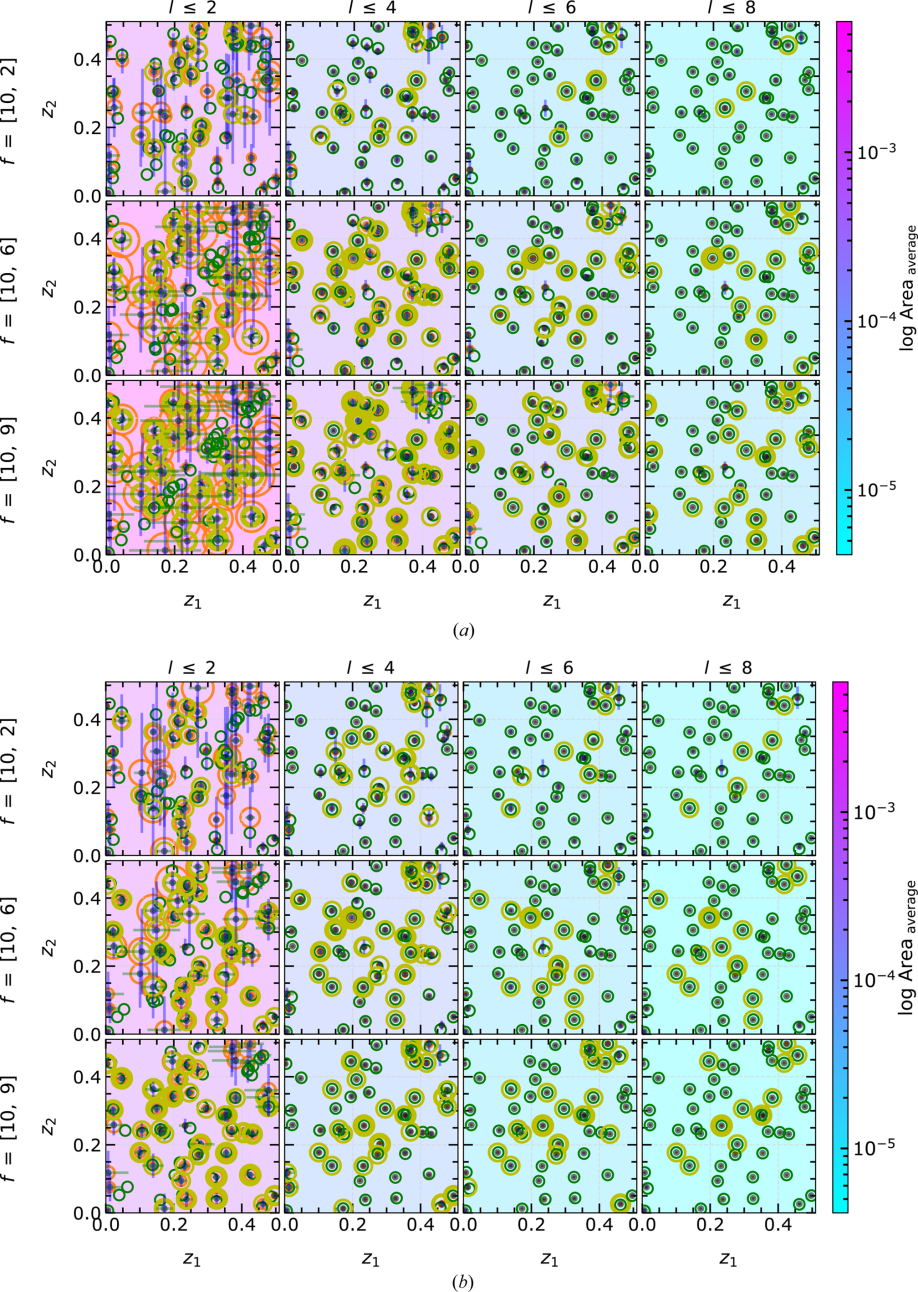
Solved atomic coordinates for (*a*) single- and (*b*) double-segment approaches using intensities within the non-EPA framework. The atomic scattering factors 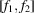 in equation (2) are set to 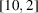 (top row), 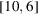 (middle row) and 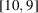 (bottom row) to represent heavy–light, heavy–medium and similar atom combinations along the 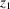 and 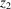 directions. The results are shown for the increasing number of reflections involved in the solution-finding process. The black dots, green circles and yellow circles (increased thickness for a larger number, *cf.* Fig. 16) represent the generated random atomic coordinates, the centroid of the solution region and the number of solution regions obtained, respectively.

The interesting question for realistic structures and diffraction data is to investigate the applicability of PSC for realistic atomic scattering factors 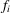, which influence the solution-finding process significantly. The results including the heavy–light atom combination 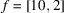 as well as the combination of similarly weighted atoms 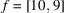 are presented in Fig. 12 ▸. For all the non-EPA cases, we focus exclusively on the more complex intensity approach, since it is the general case of experimental diffraction data with a higher multiplicity of solution regions and thus more demanding for the code.

The area of the solution region is set as the common scale across all subfigures in order to compare the effect of increasing the number of reflections in the calculation. Fig. 12 ▸ demonstrates the crucial role of the 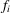 ratios in defining the size of the solution region. The different 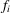 cause anisotropically deformed 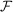 (*cf.* Fig. 6 ▸). In some cases, the linearization of such deformed 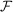 for a given *l* may contain a significantly larger area than that from other *l*. This is directly reflected in the count of observed solution regions, denoted by yellow circles in Fig. 12 ▸. Irrespective of these effects, the area of the solution region is again continuously reduced by increasing the reflection index in the calculations.

The solutions of the calculations with 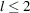 exhibit large positional uncertainties and areas, as indicated by the size of the orange circles in Fig. 12 ▸. When considering up to eight reflections, all atomic coordinates are successfully determined with positional uncertainties below 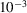 regardless of the specific combinations of 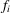. Additionally, Fig. 12 ▸ indicates that the PSC implementation effectively handles any possible combination of 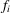 in 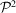. Overall, the findings demonstrate the robustness and reliability of the PSC method in accurately determining atomic coordinates, even when dealing with diverse 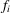 combinations.

### MC simulations in 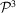


3.3.

Following the MC simulations in 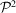, the analogous investigations are carried out in the three-dimensional PS 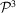. The polytopes are analyzed with their volume and extension in the 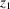, 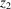 and 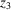 directions. Again, the solutions identified by the code are counted and their volumes are summed to construct a virtual sphere. So far, no double-segment linearization has been established for 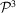, and thus we focus only on single-segment linearization. Timings and code performance for the MC simulations are evaluated in detail in Section 3.4.2.

#### 3.3.1. MC simulations within the EPA framework

**Figure 13:**
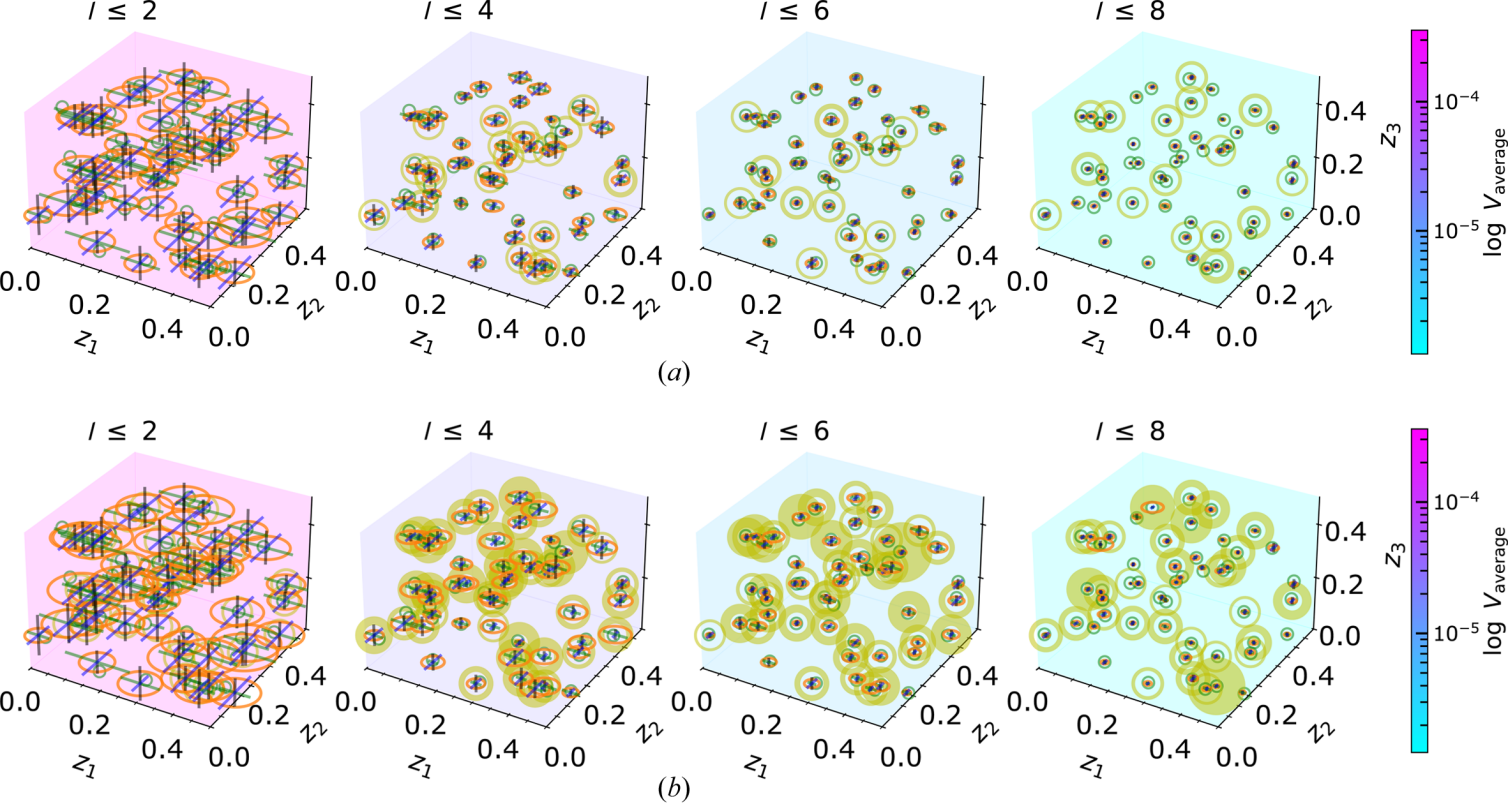
Results of MC simulations for 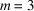 within the EPA framework. The generated atomic coordinates are solved using amplitude (*a*) and intensity (*b*). The green circle represents the centroid of the polytope that encompasses the given atomic coordinates. The cross on each point indicates the error along each 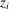 direction. When more than one solution region is available, the number is represented by the yellow circle (increased thickness for a larger number, *cf.* Figs. 17 and 18). The volume of all found solutions *V* is summed to calculate the radius of the virtual sphere as 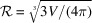, which is represented by orange circles. The background color represents the average polytope volume of all solution regions of all considered coordinates.

Fig. 13 ▸ presents the results of the MC simulations in 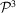 within the EPA framework, which uses both amplitude and intensity approaches. The conclusions drawn from the 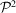 case apply similarly to 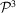. Since the single-segment linearization includes a volume around the isosurface, the intersection process potentially reveals more than one solution. By gradually adding further reflections, the positional uncertainties and the size of the solution regions are progressively reduced.

Our findings demonstrate that our code efficiently reproduces the given coordinates for the three structural degrees of freedom, regardless of the specific set of structural positions. Again, as observed in 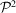, the intensity approach results in more possible solutions than the amplitude approach due to the ambiguity in the sign, and only a few solutions are identified uniquely.

#### 3.3.2. MC simulation within the non-EPA framework

**Figure 14:**
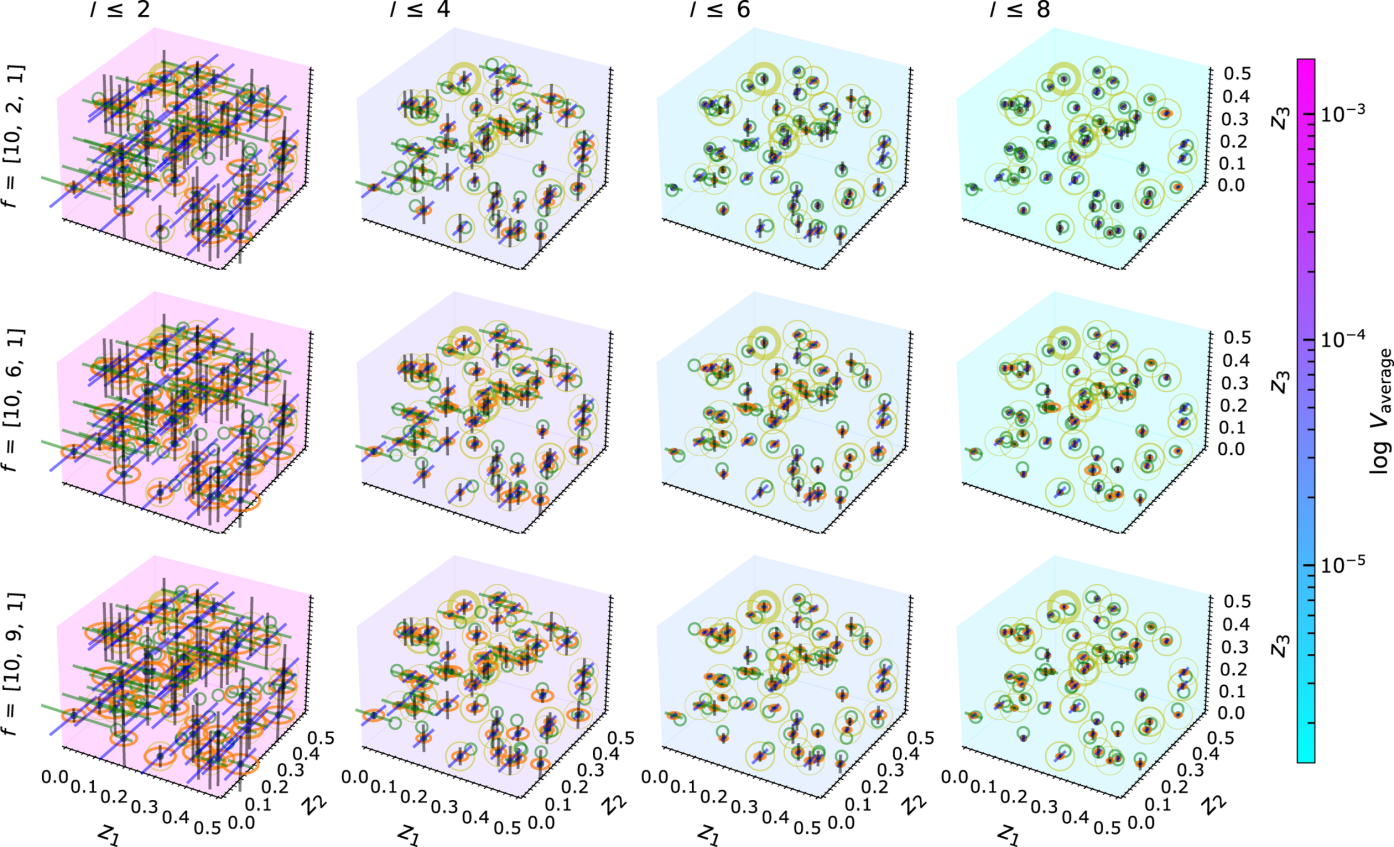
Results of MC simulations for 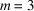 within the non-EPA framework with the intensity approach. The structures are solved for different settings of 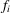. For the definition of different colors and symbols see the caption of Fig. 13 ▸.

The MC simulations are repeated for general 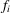 values (non-EPA) in 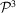. As in 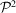 non-EPA, different combinations for atomic scattering factors are considered in 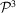, covering the three scenarios of differently weighted atoms: heavy–light–light, heavy–medium–light and heavy–heavy–light. We fixed 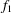 and 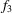 to 10 and 1, respectively, to represent heavy and light atoms, and only changed the contribution of 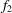. These 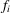 combinations are treated within the intensity approach. For comparability of the results, the initial atomic coordinates are kept identical for all scenarios; only the scattering factors 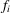 are varied (see Fig. 14 ▸).

For each scenario of 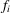 combinations, the expected reduction in volume and positional uncertainty of the solution region is visible, in analogy to the 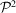 case. The variation in the 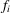 ratios affects mainly the number of solutions, as shown by the size of the virtual sphere in Fig. 14 ▸. The curvature of 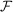 varies strongly depending on 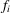 values. These multiple solutions can be further minimized or eliminated by increasing the number of reflections in the calculation and by reducing the linearization volume. If the newly considered reflection does not result in a polytope with a smaller intersection volume than the previous reflection, the intersection process will yield the same outcome as before. The presented non-EPA results demonstrate the capability of PSC in generally handling crystal systems with three structural degrees of freedom and different 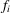 combinations, which can be utilized further for realistic diffraction data.

### 3.4. Timing benchmark

As explained in Section 2.3, the structure determination process consists of four distinct steps: initialization, linearization, intersecting and writing. We monitor the time consumption of each step to benchmark the performance of our developed algorithm. The initialization step consumes a small amount of time, taking less than a millisecond. During the linearization step, we identify the first segment, which is then repeated in the complete 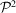/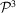 with the constraint given by equation (16), as explained in Section 2.4.5. This step consumes significantly more time. The details of the first segment are stored in a variable for later purposes. To visualize the dimensional scaling, two timings are separately captured, the time for linearization 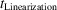 and the time required to fill the complete PS with the first segment 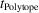 for each reflection *l*.

After linearizing and filling the PS, the subsequent intersection step is carried out to find the solution region; this step represents the most time-consuming part of the structure determination process, measured by 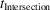. At the end, the routine creates an HDF file and writes the information about the processed reflections *l*, the first segments, found solutions, the error on each solution and the volume of each solution within the time 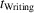. In the case of MC simulations, we additionally write the information about the generated artificial atomic coordinates and the exact solution region that encloses the given structure. The total time 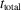 for the structure determination processes includes all four contributions separately for an increasing number of considered reflections *l*. The individual times of the MC simulation in 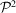 and 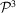 are analyzed in detail in the supporting information (Sections S3 to S6); below we only give 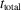.

Along with 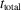, the resulting average number of solutions and maximum error on 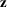 as defined in equation (17) are presented using a box plot analysis (see Section S1 for more details). Here, the average error on 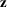 is obtained from the error on individual components, *i.e.*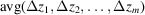, and subsequent averaging over all MC instances. In addition, the area/volume of the solution region is given by the color bar. For the purpose of readability, we display the timings for adding two consecutive reflections *l*.

#### 3.4.1. Timing benchmark for two-dimensional MC simulation

The MC results show the performance of the developed code and algorithm with respect to an increasing number of considered reflections. The individual timings are analyzed for the MC simulation in 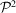 and presented in the supporting information (Sections S3 to S4) for both intensity and amplitude approaches within the EPA and non-EPA frameworks.

**Figure 15:**
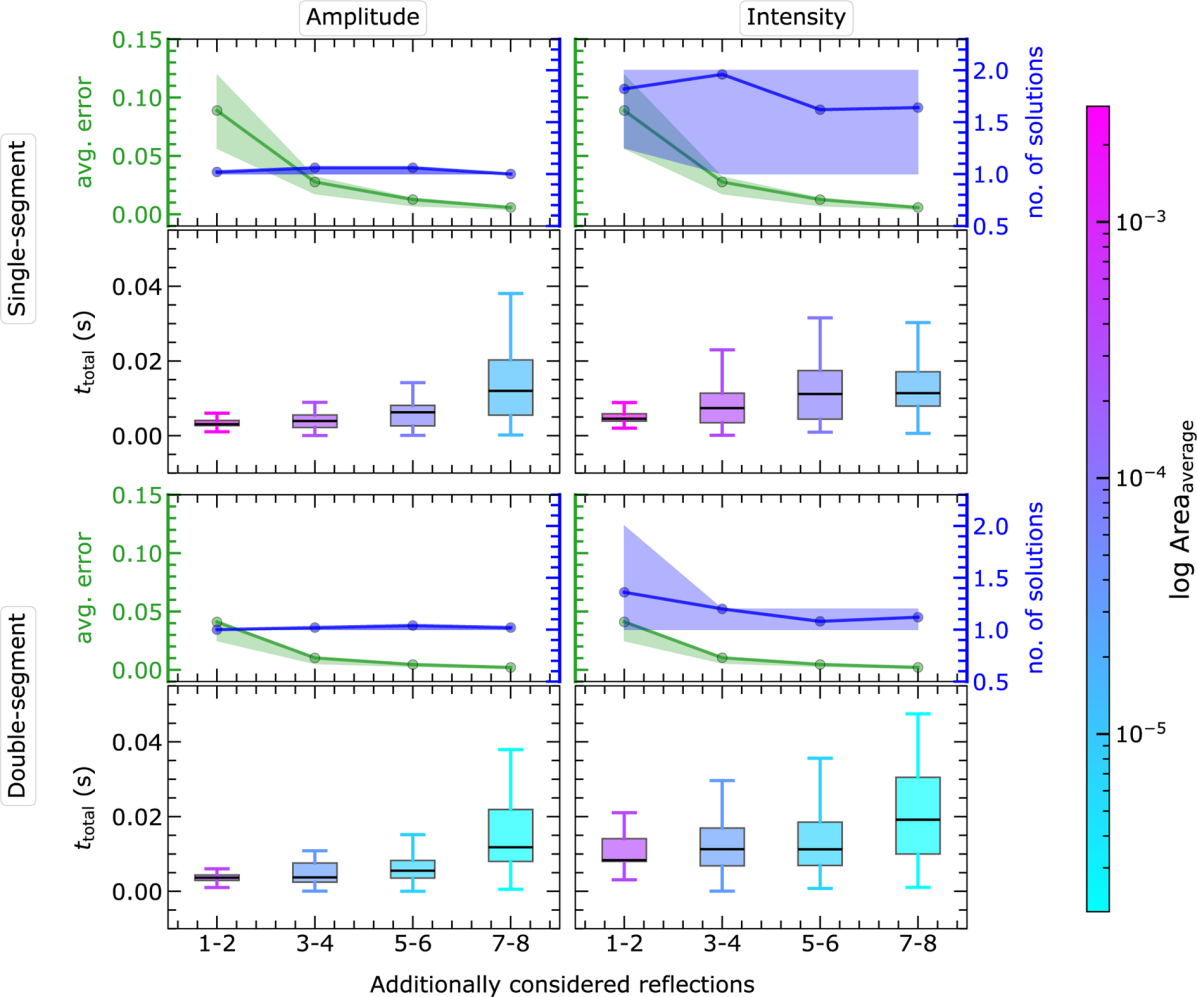
Incremental increases of 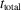 consumed for MC simulation within the EPA framework in 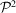, including time for linearization, polytope creation, repeating in the complete PS, intersection between successive reflections and writing found solution details to an HDF file. The timing information is presented as boxplots, highlighting the median (black horizontal line), quartiles (ends of the color box) and whiskers (here, complete data range) (for details see Section S1). In addition, the average number of solutions and average maximum possible error on computed 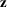 coordinates are also summarized. These two characteristics are described by the median (circle) and the whisker position (envelope). The color of the boxes represents the calculated average area of the solution regions, which can be compared with the color code given in Fig. 11 ▸. The time information for the individual process is analyzed in detail in the supporting information (see Sections S3.1 and S3.2).

As presented in Figs. 15 ▸ and 16 ▸ for EPA and non-EPA frameworks, a maximum of 12 and 200 ms, respectively, is spent solving a structure with eight reflections irrespective of 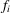 combinations. These timing observations are the result of executing the PSC code in the serial configuration. By parallelization of PSC routines, particularly the routine to fill the complete PS with segments, the time consumption can be severely reduced. Parallelization can be easily implemented in the future.

Further, the time taken for each step in the code reveals that the two parts of completing the segments in the PS and the intersection process dominate (*cf.* Sections S3 to S4). The repetition of the segment (Section 2.4.5) can also be paral­lelized, which is a future task and has not yet been implemented in the code. The computational time consumption is expected to increase following the same trends in higher dimensions.

#### 3.4.2. Timing benchmark for three-dimensional MC simulation

**Figure 17:**
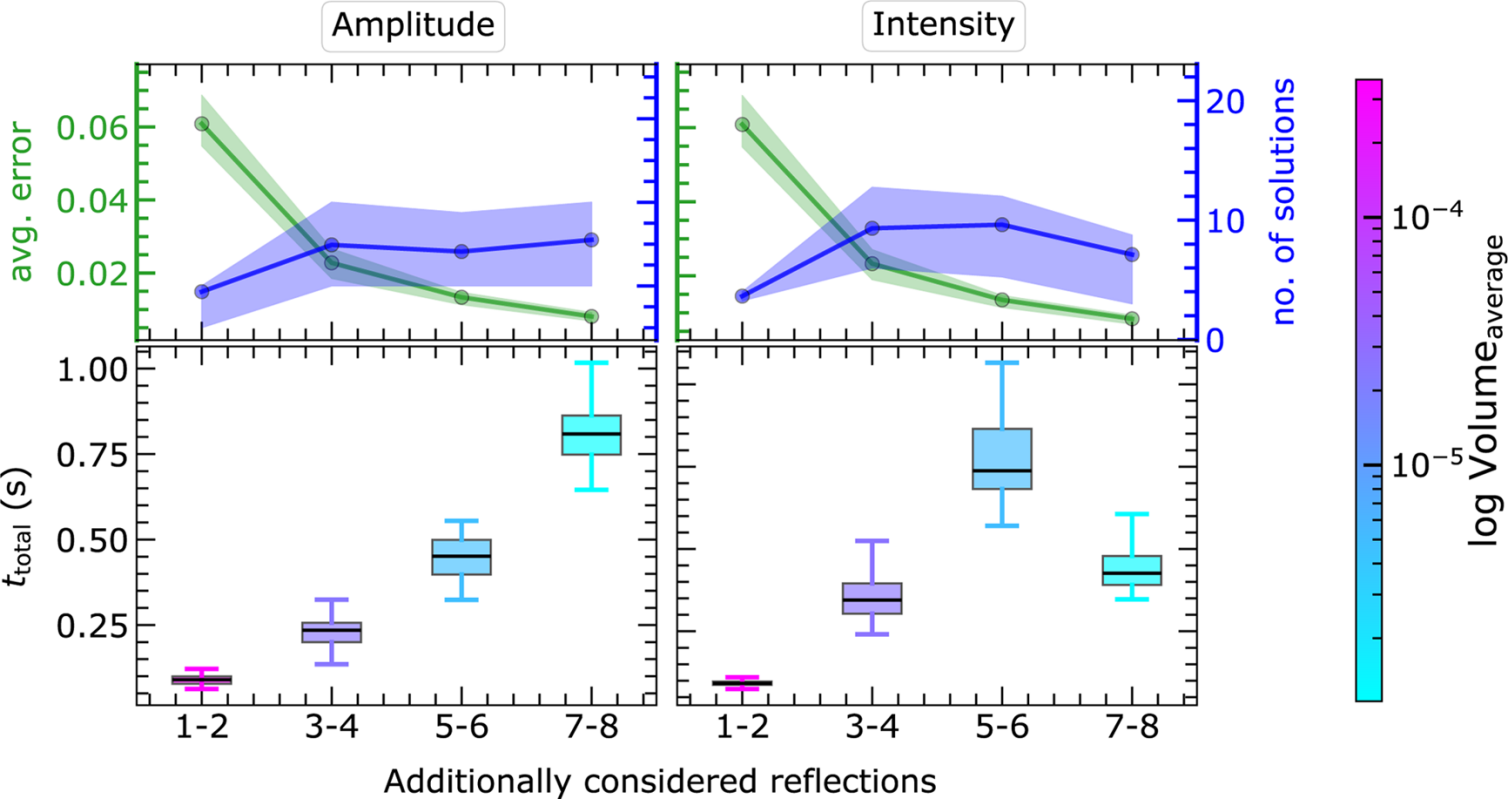
Incremental increases of 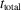 consumed in PSC simulation within the EPA framework in 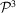, including linearization, polytope creation and repeating in the complete PS, intersection between successive reflections, and writing found solution details to an HDF file. The time information for the individual process is analyzed in detail in the supporting information (see Section S5).

As already observed for the two-dimensional cases, the repetition of segments through symmetry application and the intersection of polytopes consume more time than the linear approximation and the writing of solution details (see Sections S5 and S6). The total time for the entire process is given in Fig. 17 ▸ for the EPA framework. The structure solution within the EPA model using eight reflections takes a maximum of 10 and 2 s for the intensity and amplitude approach, respectively. The intensity approach is slower as the number of polytopes is significantly larger than for the amplitude approach.

**Figure 18:**
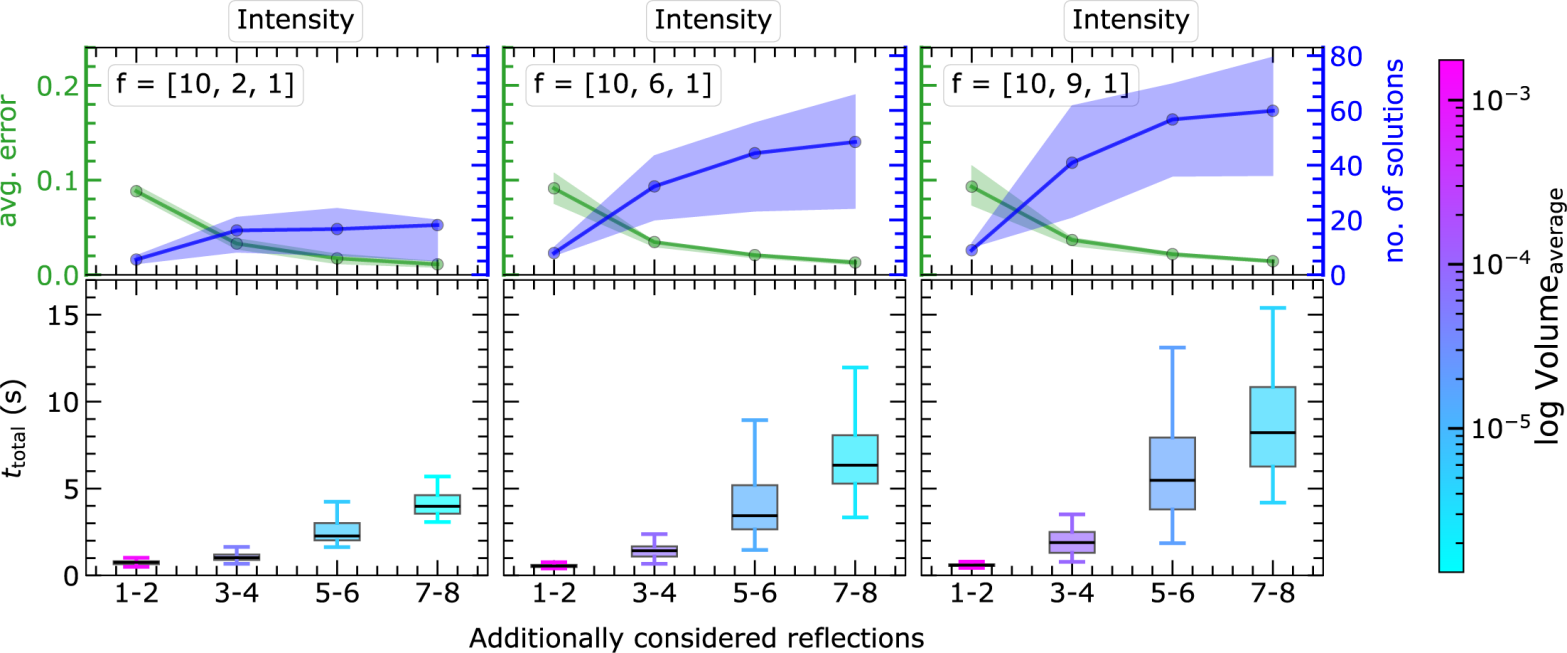
Incremental increases of 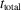 consumed in PSC simulation under the non-EPA framework in 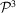, including linearization, polytope creation and repeating in the complete PS, intersection between successive reflections, and writing found solution details to an HDF file. The time information for the individual process is analyzed in detail in the supporting information (see Section S6).

In the case of the non-EPA framework, *cf.* Fig. 18 ▸, 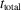 strongly varies depending on the individual structure to be solved, which is visible from the outliers of the boxplots (https://en.wikipedia.org/wiki/Box_plot). Due to the open topology, the number of possible solutions generally increases, and hence 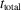 increases. Also, the computational workload increases continuously from the heavy–light–light to the heavy–heavy–light configuration.

In certain cases, the number of solutions may decrease when adding more reflections in the calculation, which may reduce the computational load. Again, for each considered structure, we observed all possible solutions in one go, *cf.* the orange spheres in Fig. 14 ▸. Hence, the PSC routines are robust for the different 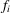 combinations in 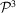.

## 4. Conclusion

Thanks to the advancements in computational resources, we are now able to apply and implement the PSC approach proposed and developed by Fischer *et al.* within the past 15 years. In the presented work, PSC has been enhanced to handle a broad spectrum of combinations of atomic scattering factors, making it suitable for realistic X-ray data analysis. In this study, a concrete workflow is developed to initialize the obtained experimental/theoretical data, linearize the amplitude or intensity, span the PS with polytopes, carry out the intersection process, and perform the solution-finding routine. A stable algorithm has been developed to linearize the amplitudes and intensities under EPA and non-EPA schemes. It is observed that the developed algorithm can handle the open topologies in isosurfaces for all general cases. So far, the developed program effectively handles the structures in 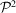 and 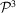.

The linearization of the isosurfaces starts with defining the inner boundary using either the intersecting points of the isosurface with each axis for closed topologies or the period for open topologies. These intersecting points are utilized by invoking the SVD method to find the required normal vector 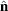. Then the respective tangent points on the isosurface are found by solving the parallel condition 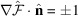 numerically using the LS method. The found tangent points are used to calculate the distance from the inner and outer boundaries to the origin. This implementation facilitates the generalization of PSC as well as the computational scaling of this step for the *m*-dimensional PS. In the next step, the required information (normal vector and boundary distances) can be converted into polytopes using efficient third-party Python libraries. The intersection process is also generalized to handle polytopes of any dimension. This step is carried out sequentially between successive reflections to scrutinize the feasible solution space for the given intensity or amplitude information.

The implemented PSC routines have been consecutively tested by employing MC simulations. Artificial atomic structures have been generated randomly and treated with EPA and non-EPA models. A comprehensive analysis is conducted in both 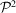 and 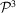, allowing not only for a visual exploration of the combined effects of different atomic scattering factors but also for qualitative analysis by evaluating the average values of area/volume of the polytopes and errors on the computed structures for each simulation step. A key observation from the simulations is that the curvature of the isosurface (*cf*. EPA versus non-EPA cases) is predominantly decreased for heavy–light atom combinations and for reflections with small structure amplitudes.

The presented results show that, with a limited number of reflections, a solution space with volume as small as 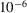 and extension on each structural degree of freedom 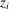 of the order of 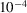 can be computed. A further advantage of PSC is that all possible structure solutions in accordance with the diffraction data appear in the PSC-determined solution volume.

In summary, the derived linearization-based PSC approach presents the means to solve crystal structures with equivalent and non-equivalent scattering factors with up to 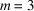 degrees of freedom in the complete PS, to handle experimental diffraction data, and to explore all possible solutions in a single analysis. Additionally, the integration of third-party Python libraries for further data post-processing introduces new strategies for determining unknown structure parameters, offering an alternative to conventional FI refinement. The routines presented here as well as informative examples are publicly available on GitHub (Vallinayagam *et al.*, 2024 ▸).

## 5. Outlook

For the generalization of the PSC routines to an *m*-dimensional PS, efficient data handling becomes crucial and calls for further development of the current methodologies. The current centrosymmetric constraint is expected to be resolved in the upcoming phase of PSC development, which should allow a further advancement in PSC code applicability.

Overall, PSC opens up new possibilities for improving the resolution and accuracy of atomic structure solutions employing resonant contrast (Zschornak *et al.*, 2024 ▸). The comparison between single- and double-segment linearization shows that precise linearization would reduce the error from linearization approximation at the cost of computational effort and the number of polytopes handled. Therefore, further development of the linearization schemes is needed.

In addition to the aforementioned challenges, the technical implementation of set theory operation critically requires major computation time. The algorithm for the intersection of polytopes is kept in step-wise operation, recursively reducing the PS with each additional reflection *l*. The themes for the next improvements are a sound parallel algorithm and innovative strategies to save the user time in handling the data. The sequence of processing the reflections may provide options to achieve the intended accuracy on atomic coordinates faster, and the search for such a processing scheme is under way. Further enhancements will increase the possible degrees of freedom and aid in handling non-centrosymmetric structures.

The implemented linearization algorithms can in principle also handle chemically complex or disordered structures, provided they maintain centrosymmetry. Point defects, including substitutional and vacancy defects, may be managed efficiently by the linearization method through variations in site occupancy and atomic scattering amplitudes [*cf*. equation (2) and Figs. 12 ▸ and 14 ▸ for the effects on structure determination due to varying atomic scattering amplitudes]. Interstitial defects can be addressed by treating the defect site as an additional degree of freedom. However, displacive and dynamic disorder requires consideration of a span of atomic positions, presenting more challenges that are beyond the scope of the current PSC routines. Well ordered extended defects may be modeled using supercells with rational modulation vectors, requiring further degrees of freedom depending on the structural details. Overall, addressing these defect scenarios will be a task for future work and is not part of the present study.

Moreover, predicting the exact structure from multiple possible solutions is an open issue. A significant number of non-unique solutions will disappear once the sign of intensity is known and utilized. More may be identified as non-valid with sufficiently small intensity errors or the use of resonant contrast. The linearization method leverages the advantages of modern synchrotron X-ray sources, particularly through the use of multiple wavelengths and identified phases of the amplitudes, along with high-quality intensities [narrow isosurfaces, see *e.g.* Figs. 4–6 of Zschornak *et al.* (2024 ▸)] as well as high resolution in reciprocal space and high *q* values. In any case, the remaining non-unique solutions may be further analyzed and refined with modern state-of-the-art theoretical simulations like density functional theory (Blöchl, 1994 ▸; Kresse & Joubert, 1999 ▸; Perdew *et al.*, 1996 ▸) calculations, where the ground-state energies of the predicted structures can be assessed to discriminate chemically unstable solutions.

In the present implementation, all routines are in principle generalized in such a way that they can be extended to handle arbitrary non-centrosymmetric structures in *m*-dimensional PS. Performance and reliability tests for 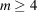 are currently in progress and will be the scope of further work.

## 6. Related literature

The following references are cited in the supporting information: Komorowski *et al.* (2008 ▸), Rousseeuw *et al.* (1999 ▸), Benjamini (1988 ▸); *Matplotlib* (https://matplotlib.org/stable/api/_as_gen/matplotlib.pyplot.box.html, https://www.tutorialspoint.com/matplotlib/matplotlib_box_plot.htm).

## Supplementary Material

Supporting information, providing additional results. DOI: 10.1107/S1600576725001955/yr5148sup1.pdf

## Figures and Tables

**Figure 16:**
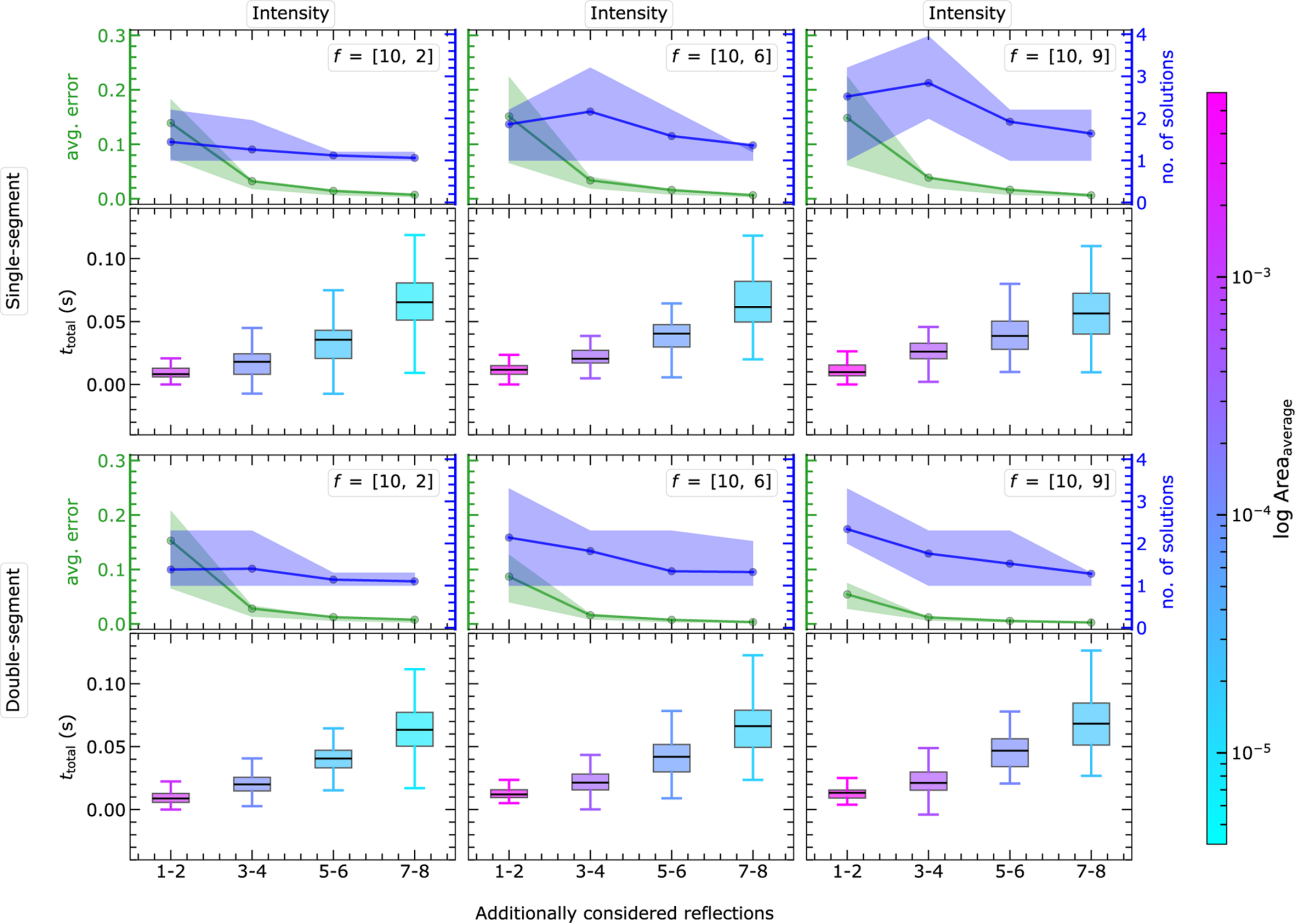
Incremental increases of 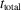 consumed for MC simulation within the non-EPA framework in 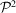. The color of the boxes represents the calculated average area of solution regions which can be compared with the color code given in Fig. 12 ▸. For further explanation see the caption of Fig. 15 ▸. The time information for the individual process is analyzed in detail in the supporting information (see Sections S4.1 and S4.2).

**Table 1:** Comparison of theoretical (from the atomic structure) and calculated (from the tangent point) intensities for the atomic structure 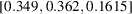 The found normal vector 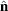, the tangent point for the given atomic scattering factors 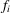 and the deviation angles are also listed. A large value of the difference in angle indicates a larger deviation. The tangent point column lists two sets of coordinates in two rows corresponding to the inner (first row) and outer (second row) boundaries. The respective 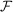
s along with the listed tangent points (filled black points) are given in Fig. 7 ▸.

		*I*				
Reflection	*f*	Theoretical	Calculated	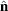	Tangent points	Deviation angle (°)
6	[10, 9, 4]	259.427	259.427	[0.680, 0.639, 0.358]	[0.000, 0.000, 0.063]	0.0005
					[0.018, 0.018, 0.027]	0.0005
1	[10, 7, 1]	100.431	100.431	[0.777, 0.626, 0.055]	[0.000, 0.226, 0.500]	0.0005
					[0.144, 0.179, 0.093]	0.0005
8	[10, 1, 1]	63.042	63.042	[0.992, 0.088, 0.088]	[0.002, 0.060, 0.060]	0.0004
					[0.017, 0.015, 0.015]	0.0004
9	[10, 1, 1]	0.68	0.68	[0.996, 0.064, 0.064]	[0.023, 0.044, 0.044]	0.0004
					[0.029, 0.012, 0.012]	0.0004
