# Supplementary material for: Linearization routines for the parameter space concept to determine crystal structures without Fourier inversion
Source: J Appl Crystallogr. 2025 May 23;58(Pt 3):768–88. doi: 10.1107/S1600576725001955 (PMC12135979; doi:10.1107/S1600576725001955)
Supplement: Supplementary file 1 [file j-58-00768-sup1.pdf]

JOURNAL OF APPLIED CRYSTALLOGRAPHY

Volume 58 (2025)

Supporting information for article:

Linearization routines for the parameter space concept to determine crystal structures without Fourier inversion

Muthu Vallinayagam, Melanie Nentwich, Dirk C. Meyer and Matthias Zschornak

1. General remarks on time information

In the following sections, the individual time consumed in each Monte-Carlo (MC) simulation is presented. Also, the average error found on each z_i coordinate and the average number of solutions is presented similarly to the publication for easy comparison. The individual time information includes time spent for linearization, polytope creation in the entire parameter space (PS), and writing the solution in an HDF file. The total time for all processes is summed and presented.

Overall, it is understandable that the major time is spent during the polytope creation and intersection steps. As mentioned in the publication, for the EPA framework, the f_i's are set to 1 and for the non-EPA framework, different f_i combinations are assumed. A total of 50 random artificial structures were generated and solved with the current implementation. For each coordinate a set of time information is saved, which provides a solid database for statistical analysis by means of *Box Plots* [1, 2, 3].

A box plot is a diagram to visualize a variety of statistical values in one display in order to give a quick overview of how the data distributes. The statistical measures are described using the five-number summary including the median, upper and lower quartile as well as upper and lower whiskers, see SFig. 1. The median is defined as the middle value of the data set; it marks the value at which 50 % of the data is both above and below it. The quartiles mark those data points where 25 % (a quarter) of the data is above or below, respectively. The lower and upper quartiles (Q_1 and Q_3) mark the borders of the box, which comprises 50 % of the data, by definition. The span of the box is the *Inter-Quartile Range* $\mathrm{IQR} = Q_3 - Q_1$. In contrast to those well-defined values, the position of the whiskers is more flexible and can be adapted to the data sets under investigation. In general, they define, which data points are considered outliers and represent observed data points. Here, we use the following definition for the whiskers: $Q_1 - x \cdot \mathrm{IQR}$ and $Q_3 + x \cdot \mathrm{IQR}$, with the default value $x = 1.5$, as recommended by the matplotlib guideline from Python [2]. More details can be found in Refs. [4, 5, 6].

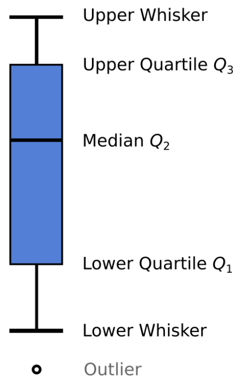

SFig. 1: Schematic drawing of a box plot, highlighting its five-number summary (black labels). The five numbers include median, lower, and upper quartile as well as lower and upper whisker.

2. HDFView window

The HDF files can be viewed using HDFView software [7]. A single HDF file can contain multiple data types. The file itself acts as the root directory including so-called *objects*. Within objects, any number of data can be stored. SFigure 2 shows an example of the home window in HDFView,

with an open file named *example_HDF.h5*. It contains the objects `allsolution`, `error`, `extreme`, `generatedcoordinate`, `polytope`, `total_volume_in_Asym`, `unsortedcoordinate`, and `vol`. More information is available in the *General Object Info* tab of the main window. A simple click on the data can open it in a separate window. As an example, the data entry *v0* is a 64-bit floating data with a value 7.49×10^{-4}, highlighted by the yellow box.

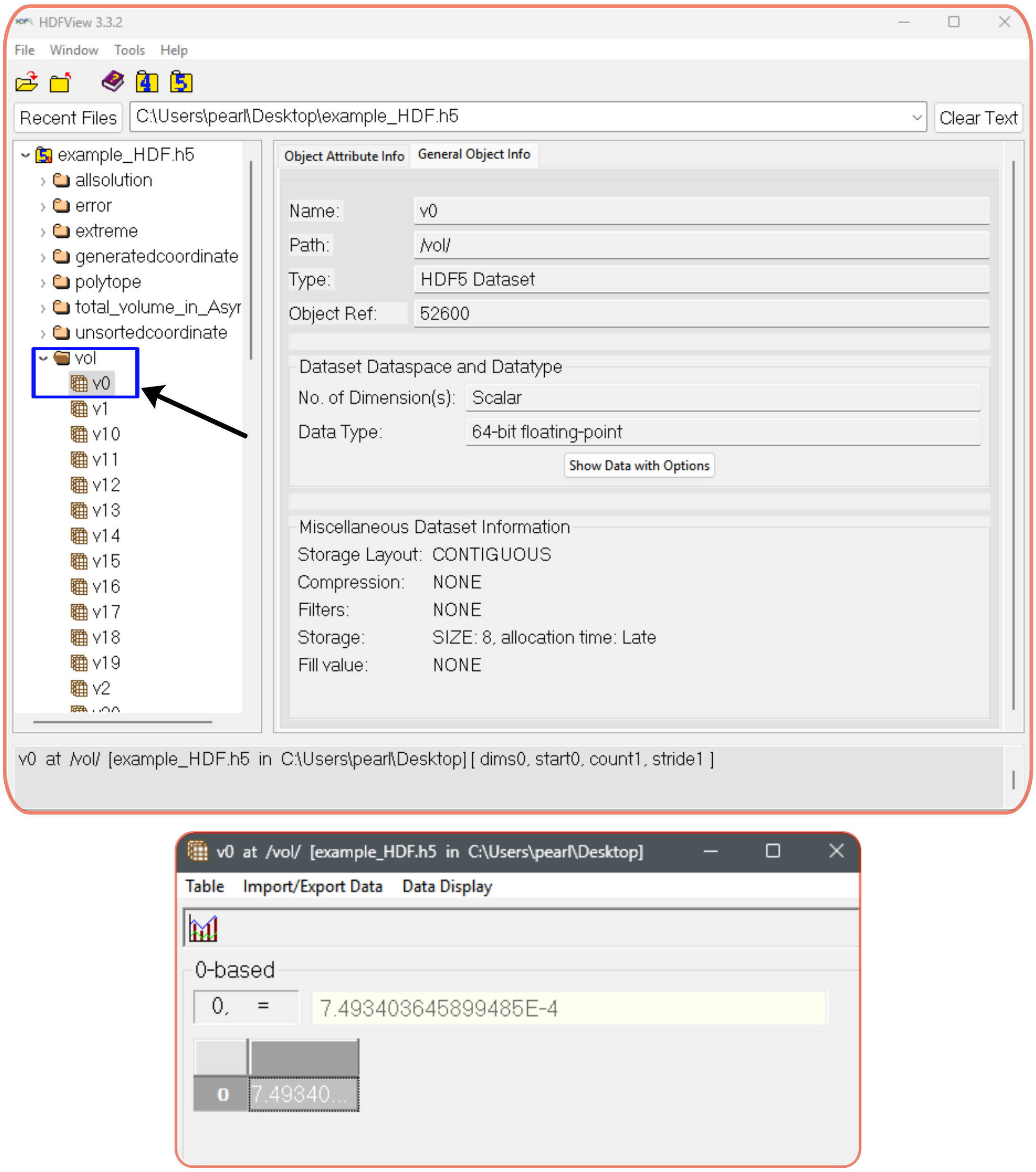

SFig. 2: An example HDF file is opened using the HDFView software. The object entry `vol`, highlighted by a blue box, is expanded. The data entry *v0* is selected and its detail is shown in the bottom window.

3. Timing benchmark for individual steps of structure determination in $\mathcal{P}^2$ under the EPA framework

As discussed in the main article, individual times in the complete structure determination process are analyzed using box plots. In the underlying *Monte Carlo* (MC) simulations, we considered the reflections 1 to 8. For better visibility and simplicity, we show the time analysis in steps of 2 additionally considered reflections. Each of the steps considers the linearization and polytope creation of two reflections, 1 and 2, 3 and 4, and so on. Additionally, the time for intersecting and writing is added to the total timing. For the first step, the intersecting process only occurs once (between reflections 1 and 2), just like the solution writing. After that, the intersecting process is performed twice (between the previous solution regions and the first new reflection and with the second new reflection), and both results are written independently. Therefore, the required time might only show a functional dependence after the first step.

3.1 EPA single segment MC simulation in $\mathcal{P}^2$

The time information for the MC simulations in $\mathcal{P}^2$, performed within the single-segment (SS) EPA framework with both amplitude and intensity treatments, is presented in SFig. 3. A comparison between amplitude and intensity treatments indicates that the intensity approach introduces greater ambiguity of the solutions, leading to a higher number of solutions than the amplitude approach. This is reflected in the slightly higher time consumption for t_{polytope} of the intensity approach, which in general follows an exponential increase with increasing order of l. The time taken by other processes remains fairly similar across both approaches. The time required for writing the results is relatively constant, independent of the considered reflections, not only for the constraints in this section, but for all constellations analyzed within this supplement.

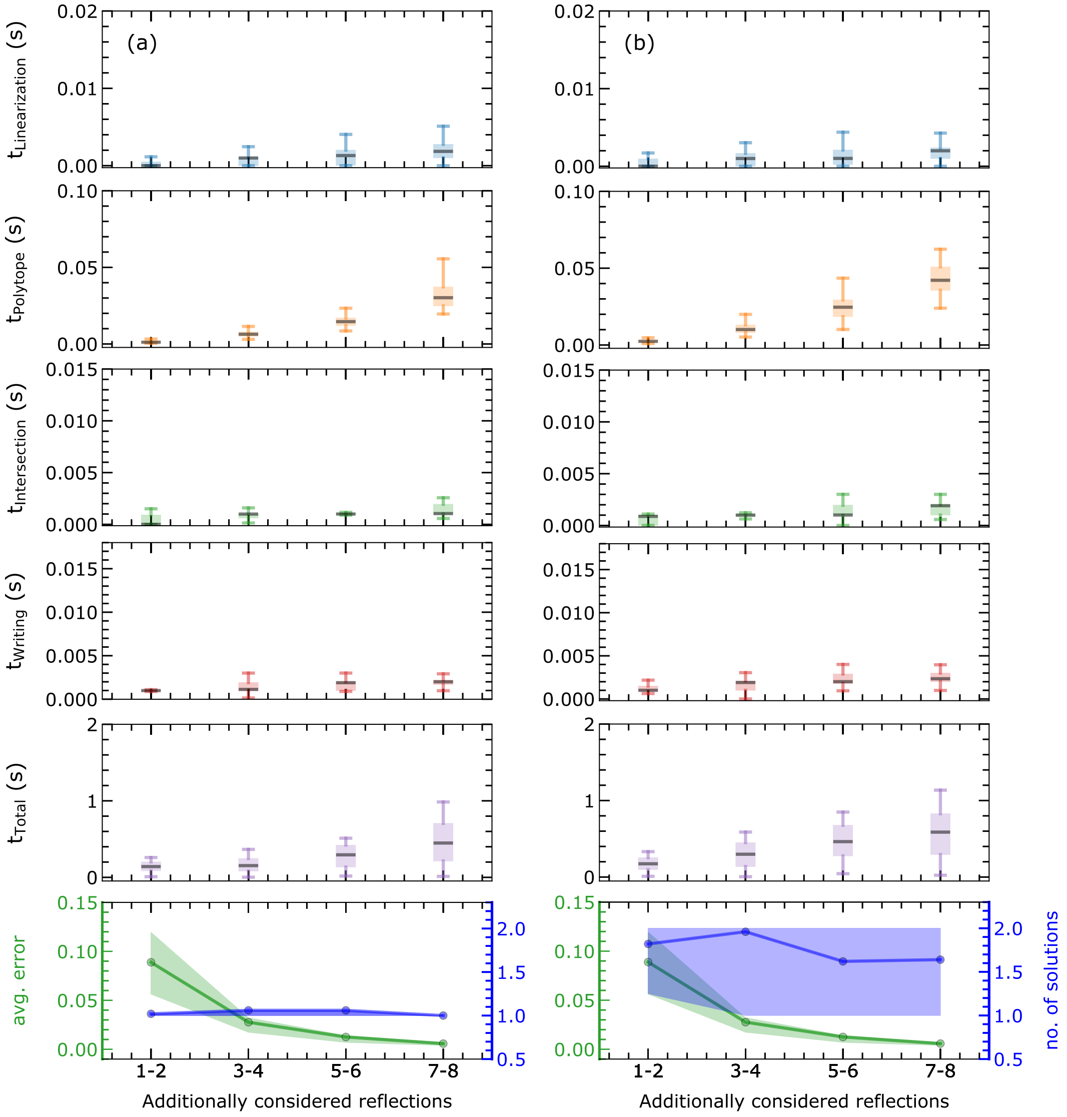

SFig. 3: The timing benchmark for the MC simulations in $\mathcal{P}^2$ under the EPA framework, which uses the single segment approach. The structures are solved with (a) amplitudes and (b) intensities of the given structure for various reflections. The number of considered reflections is given on the x-axis. The timing for the number 3 to 4, for example, represents the time consumed to process the reflections 3 and 4.

3.2 EPA double segment MC simulation in $\mathcal{P}^2$

The time information for the MC simulations in $\mathcal{P}^2$ under the double-segment (DS) EPA framework with amplitude and intensity treatment is shown in SFig. 4. Since the segment in the DS approach is smaller than in the SS approach, higher accuracy of the solution is obtained. In comparison, the double-segment provides higher resolution by a factor of 2.7 with just the first two reflections and 3.6 with 8 reflections. The comparison between amplitude and intensity treatment reveals that for the median calculation the t_{polytope} consumes about twice as much time for intensity treatment for all reflection steps due to the larger number of polytopes involved in the calculation. Also, the intensity approach includes more ambiguity, resulting in more solutions than the amplitude approach. By the computational load up to $2\,s$, the DS results in a more accurate solution than the SS approach.

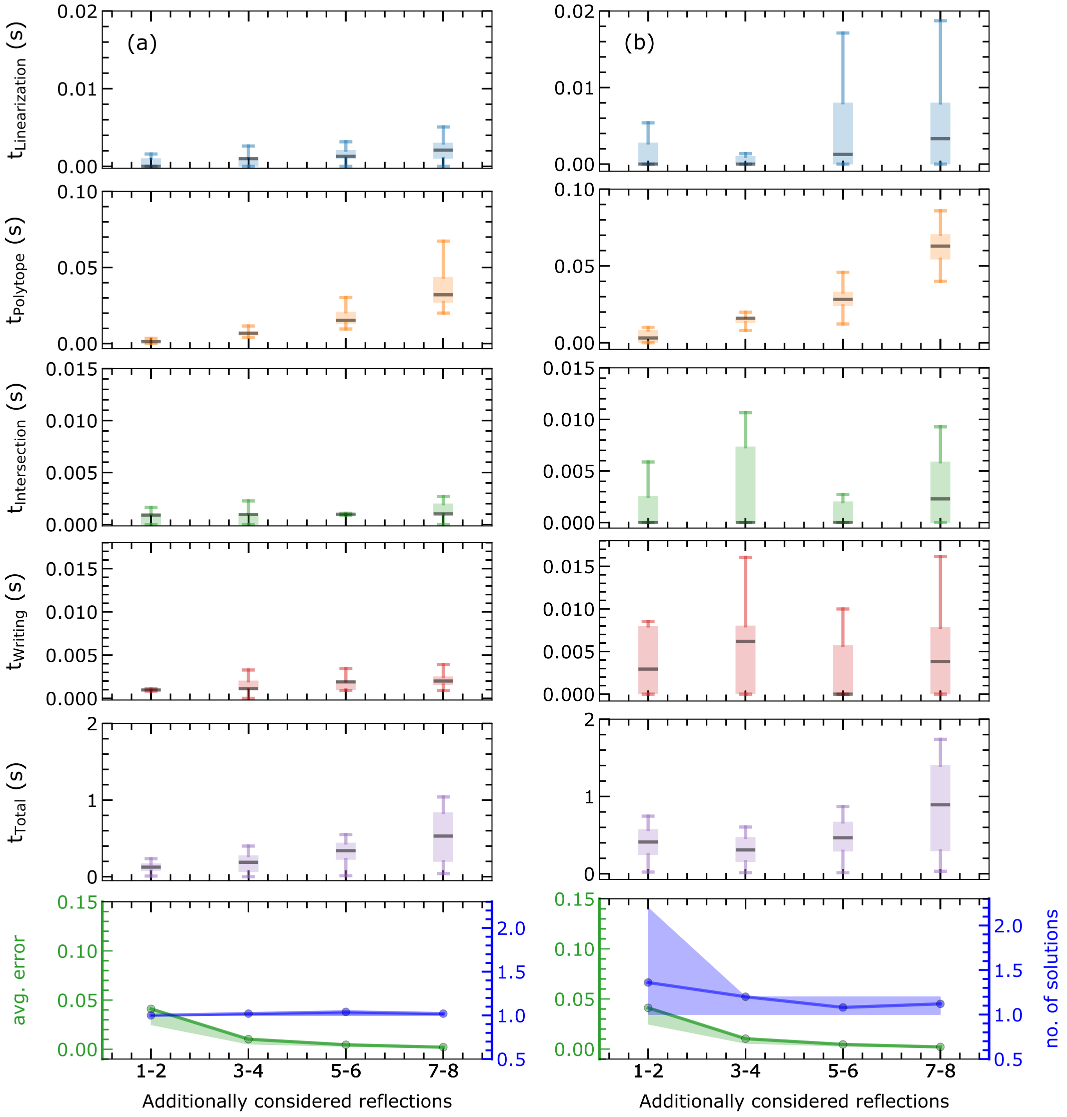

SFig. 4: The timing benchmark for the MC simulations in $\mathcal{P}^2$ under the EPA framework, which uses the double segment approach. The structures are solved with (a) amplitudes and (b) intensities of the given structure for various reflections. The number of considered reflections is given on the x-axis. The timing for the number 3 to 4, for example, represents the time consumed to process the reflections 3 and 4.

4. Timing benchmark for individual steps of structure determination in $\mathcal{P}^2$ under the non-EPA framework

4.1 non-EPA single segment MC simulation in $\mathcal{P}^2$

The effect of f_i combination on individual timings within the SS approach is shown in Fig. 5. Again, it is inferred that the t_{polytope} is considerably higher than other timings irrespective of f_i combinations. However, within t_{total} of 4 s, less than 4 different solution regions are found by the linearization approximation.

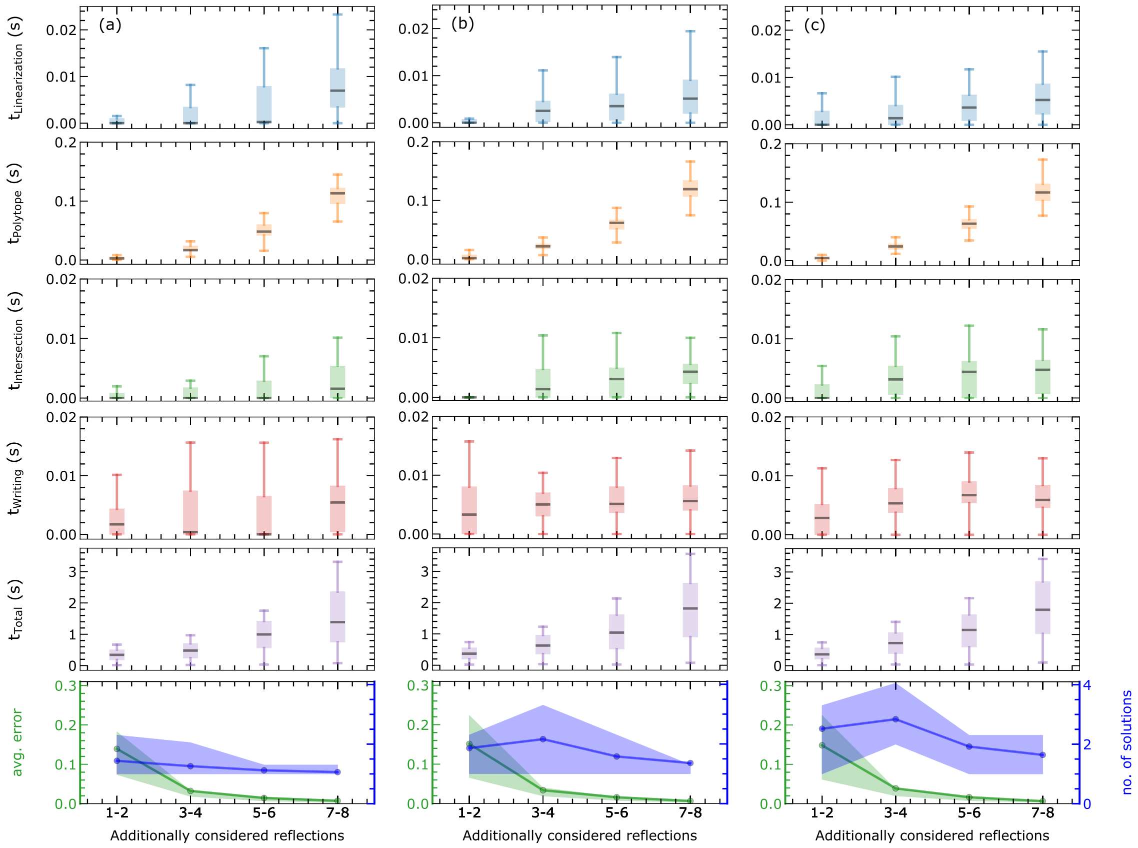

SFig. 5: The timing benchmark for the MC simulations in $\mathcal{P}^2$ under the non-EPA framework, which uses the single segment approach. The structures are solved using the atomic structure factor combinations (a) $f = [10, 2]$, (b) $f = [10, 6]$, and (c) $f = [10, 9]$. The intensities of the given structure are assumed within the process. The number of considered reflections is given on the x-axis. The timing for the number 3 to 4, for example, represents the time consumed to process the reflections 3 and 4.

4.2 non-EPA double segment MC simulation in $\mathcal{P}^2$

The effect of f_i combination on individual timings within the DS approach is shown in Fig. 6. Again, it is inferred that the t_{polytope} is considerably higher than other timings irrespective of f_i combinations. However, within t_{total} of 4 s, up to 3 different solution regions are found by the linearization approximation. Like before, as the reflection l increases, the accuracy on determined structures is increasing.

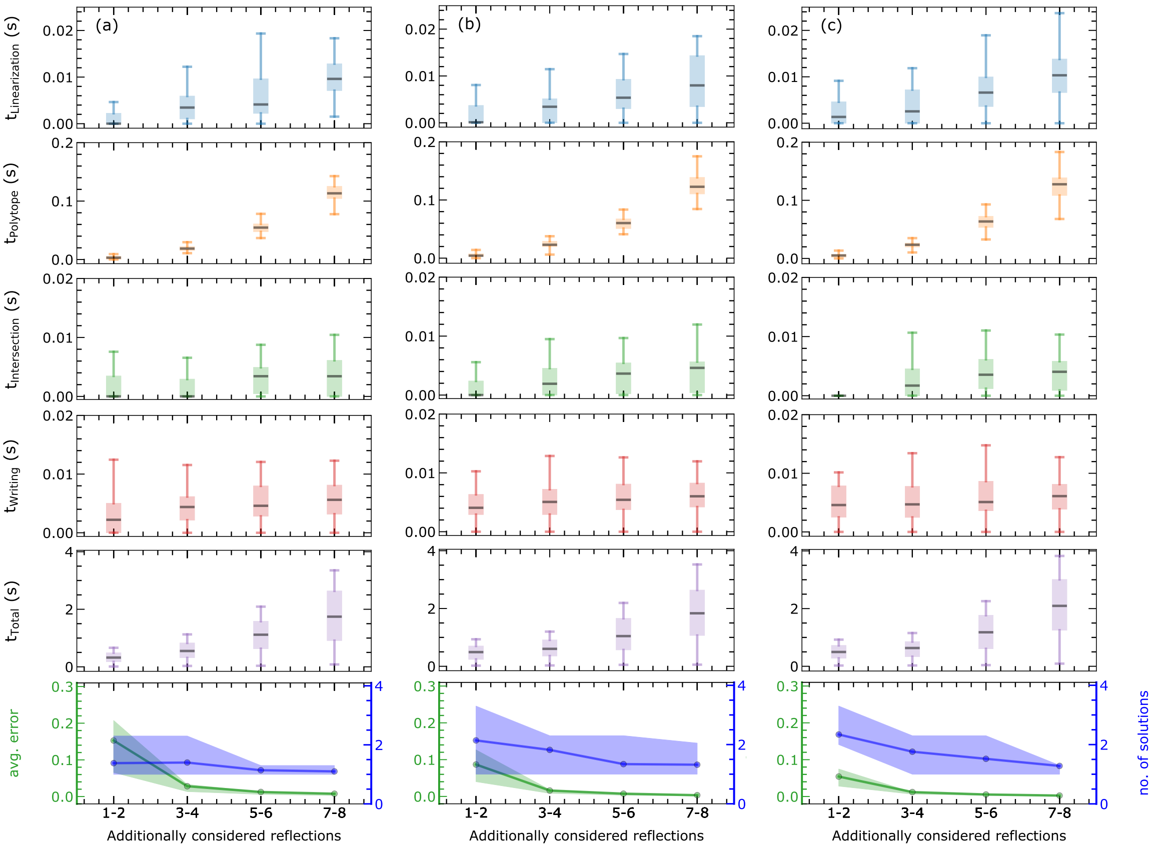

SFig. 6: Timing benchmark for the MC simulations in $\mathcal{P}^2$ under the non-EPA framework, which uses the double segment approach. The structures are solved using the atomic structure factor combinations (a) $f = [10, 2]$, (b) $f = [10, 6]$, and (c) $f = [10, 9]$. The intensities of the given structure are assumed within the process. The intensities of the given structure are assumed within the process. The number of considered reflections is given on the x-axis. The timing for the number 3 to 4, for example, represents the time consumed to process the reflections 3 and 4.

5. Timing benchmark for individual steps of structure determination in $\mathcal{P}^3$ under the EPA framework

5.1 EPA MC simulation in $\mathcal{P}^3$

The intensity and amplitude treatment are used in solving the structures within the EPA framework. Again, it is found that the amplitude approach provides better resolution on the identified solution region. The t_{total} for intensity and amplitude approaches differ significantly by a factor of 3. The intensity treatment provides more ambiguous solutions than the amplitude treatment. The significant reduction in computational load for the intensity approach seen in all timings may happen whenever the cross-section of the intermittent solution regions with a newly added, linearized isosurface cancels out a large number of polytopes.

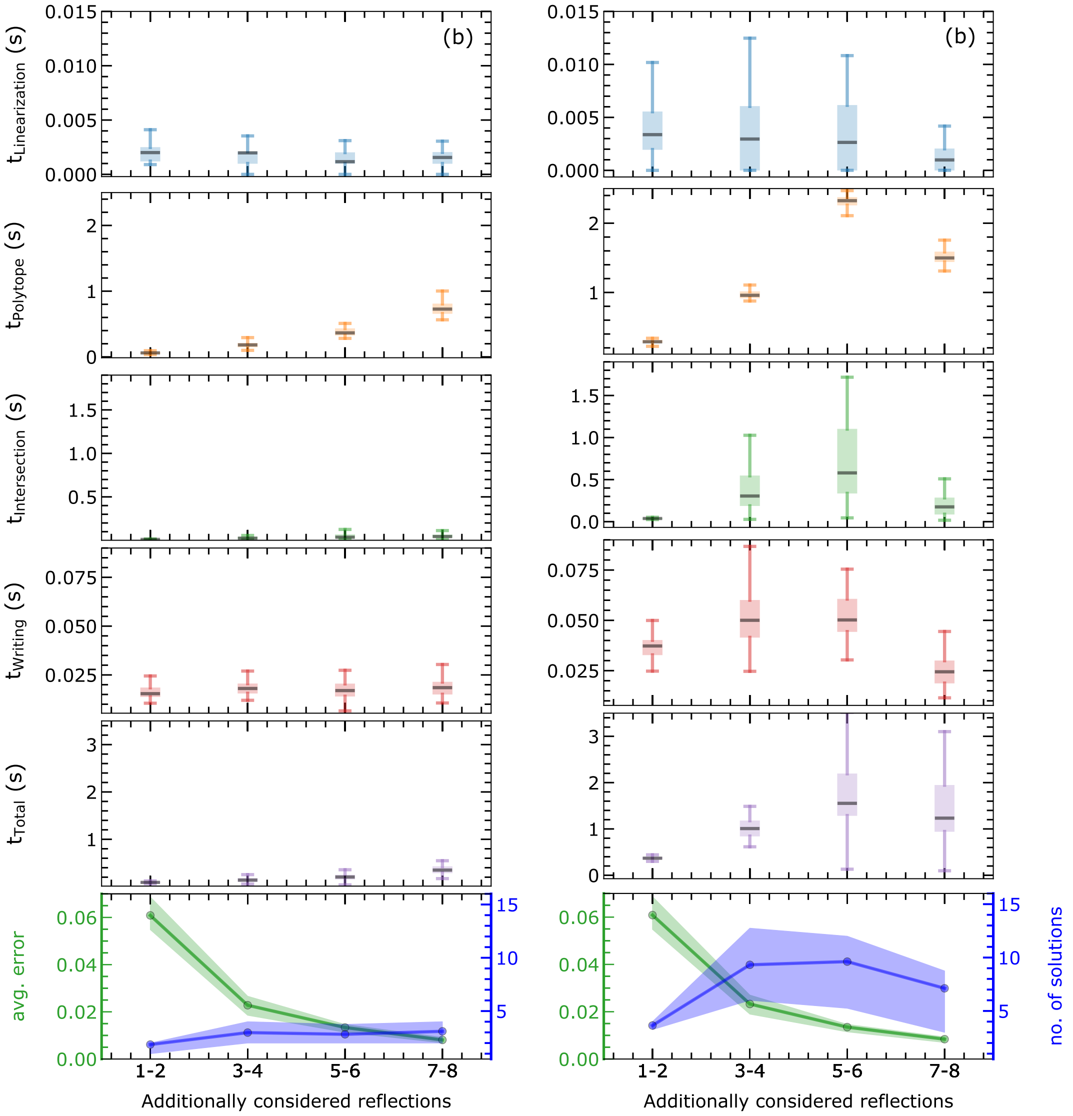

SFig. 7: Timing benchmark for the MC simulations in $\mathcal{P}^3$ under the EPA framework. The structures are solved with (a) amplitudes and (b) intensities of the given structure for various reflections. The intensities of the given structure are assumed within the process. The number of considered reflections is given on the x-axis. The timing for the number 3 to 4, for example, represents the time consumed to process the reflections 3 and 4.

6. Timing benchmark for individual steps of structure determination in $\mathcal{P}^3$ under the non-EPA framework

6.1 non-EPA MC simulation in $\mathcal{P}^3$

The f_i combination has a predominant effect on individual timings as the amount of considered reflections increases within the non-EPA approach. The structures are solved using intensity values. When considering two atoms with medium or high weight in SFig. 8(b) and (c), the number of solution regions increases. Better accuracy on the found solution is achieved by including more reflections in the calculation, as has been evident in all calculations.

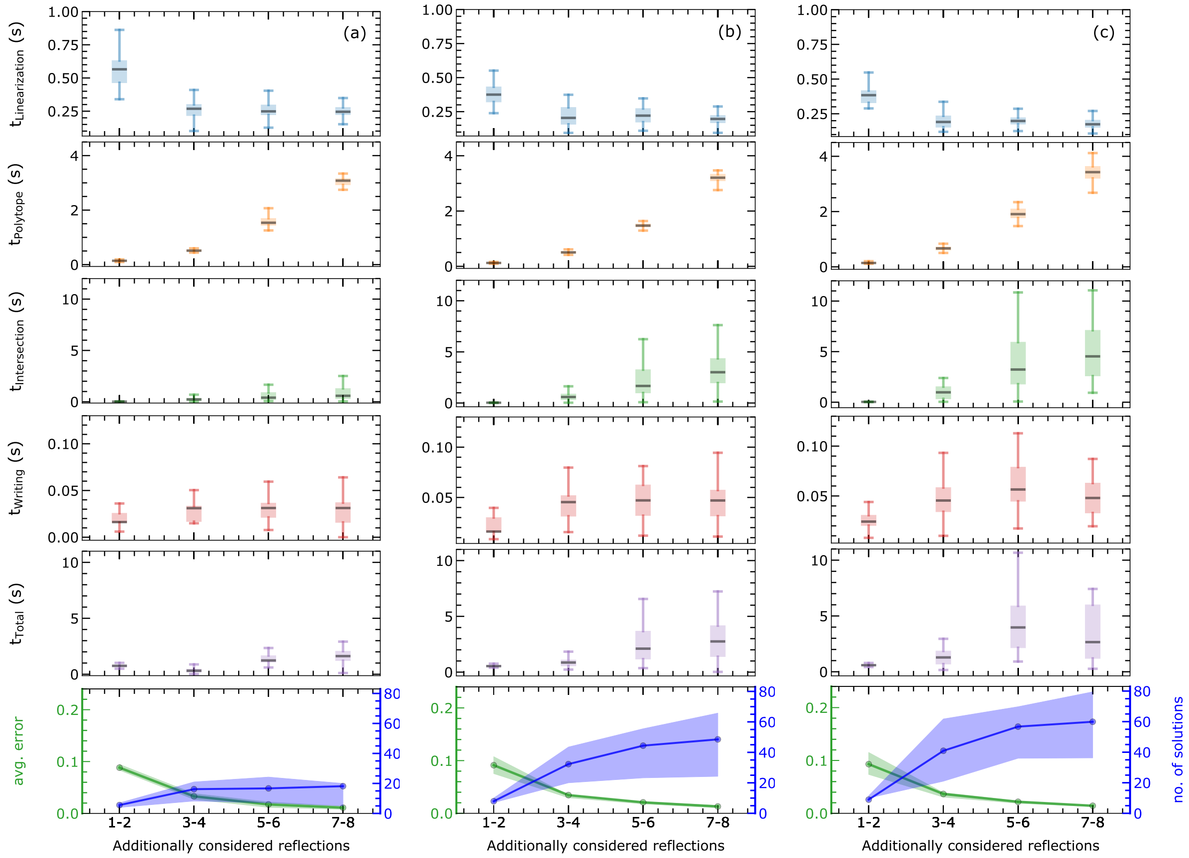

SFig. 8: Individual timing benchmark for the MC simulations in $\mathcal{P}^3$ under the non-EPA framework. The structures are solved using the atomic structure factor combinations (a) $f = [10, 2, 1]$, (b) $f = [10, 6, 1]$, and (c) $f = [10, 9, 1]$. The intensities of the given structure are assumed within the process. The intensities of the given structure are assumed within the process. The number of considered reflections is given on the x-axis. The timing for the number 3 to 4, for example, represents the time consumed to process the reflections 3 and 4.

References

[1] *Box plot from Wikipedia*, https://en.wikipedia.org/wiki/Box_plot.

[2] *Matplotlib documentation*, https://matplotlib.org/stable/api/_as_gen/matplotlib.pyplot.boxplot.html.

[3] *Matplotlib documentation*, https://www.tutorialspoint.com/matplotlib/matplotlib_box_plot.htm#:~:text=It%20displays%20the%20summary%20statistics,the%20minimum%20and%20maximum%20values.

[4] *Exploratory Data Analysis*, Springer New York, New York, NY, 2008, p. 192.

[5] P. J. Rousseeuw, I. Ruts and J. W. Tukey, The Bagplot: A Bivariate Boxplot, *Am. Stat.*, 1999, ***53***, *382*.

[6] Y. Benjamini, Opening the Box of a Boxplot, *Am. Stat.*, 1988, ***42***, *257*.

[7] T. H. Group and Q. Koziol, *HDF5-Version 1.12.0*, 2020, https://doi.org/10.11578/dc.20180330.1.
